# Morphological, Structural, and Functional Networks Highlight the Role of the Cortical-Subcortical Circuit in Individuals With Subjective Cognitive Decline

**DOI:** 10.3389/fnagi.2021.688113

**Published:** 2021-07-09

**Authors:** Xiaowen Xu, Tao Wang, Weikai Li, Hai Li, Boyan Xu, Min Zhang, Ling Yue, Peijun Wang, Shifu Xiao

**Affiliations:** ^1^Department of Medical Imaging, Tongji Hospital, Tongji University School of Medicine, Tongji University, Shanghai, China; ^2^Department of Geriatric Psychiatry, Shanghai Mental Health Center, Shanghai Jiao Tong University School of Medicine, Shanghai, China; ^3^Alzheimer’s Disease and Related Disorders Center, Shanghai Jiao Tong University, Shanghai, China; ^4^College of Computer Science and Technology, Nanjing University of Aeronautics and Astronautics, Nanjing, China; ^5^Center for MRI Research, Academy for Advanced Interdisciplinary Studies, Peking University, Beijing, China; ^6^McGovern Institute for Brain Research, Peking University, Beijing, China; ^7^Beijing Intelligent Brain Cloud Inc., Beijing, China

**Keywords:** subjective cognitive decline, morphological network, structural network, functional network, multiple kernel learning

## Abstract

Subjective cognitive decline (SCD) is considered the earliest stage of the clinical manifestations of the continuous progression of Alzheimer’s Disease (AD). Previous studies have suggested that multimodal brain networks play an important role in the early diagnosis and mechanisms underlying SCD. However, most of the previous studies focused on a single modality, and lacked correlation analysis between different modal biomarkers and brain regions. In order to further explore the specific characteristic of the multimodal brain networks in the stage of SCD, 22 individuals with SCD and 20 matched healthy controls (HCs) were recruited in the present study. We constructed the individual morphological, structural and functional brain networks based on 3D-T1 structural magnetic resonance imaging (sMRI), diffusion tensor imaging (DTI) and resting-state functional magnetic resonance imaging (rs-fMRI), respectively. A *t*-test was used to select the connections with significant difference, and a multi-kernel support vector machine (MK-SVM) was applied to combine the selected multimodal connections to distinguish SCD from HCs. Moreover, we further identified the consensus connections of brain networks as the most discriminative features to explore the pathological mechanisms and potential biomarkers associated with SCD. Our results shown that the combination of three modal connections using MK-SVM achieved the best classification performance, with an accuracy of 92.68%, sensitivity of 95.00%, and specificity of 90.48%. Furthermore, the consensus connections and hub nodes based on the morphological, structural, and functional networks identified in our study exhibited abnormal cortical-subcortical connections in individuals with SCD. In addition, the functional networks presented more discriminative connections and hubs in the cortical-subcortical regions, and were found to perform better in distinguishing SCD from HCs. Therefore, our findings highlight the role of the cortical-subcortical circuit in individuals with SCD from the perspective of a multimodal brain network, providing potential biomarkers for the diagnosis and prediction of the preclinical stage of AD.

## Introduction

Alzheimer’s Disease (AD) is the most common cause of dementia, characterised by irreversible neurodegeneration and continuous cognitive function decline (Bonte et al., 1986; Scheltens et al., 2016). It is generally believed that the early diagnosis of AD is crucial for early intervention and improving the therapeutic effects of AD treatment. Subjective cognitive decline (SCD) is considered the earliest stage of the clinical manifestations of progressively developing AD (Jessen et al., 2014a, 2020). Thus, SCD is valuable for the early diagnosis and prediction of AD.

Multimodal neuroimaging studies have indicated that individuals with SCD show a greater similarity to AD in their patterns of brain structure and function compared with healthy controls (HCs) (Lin et al., 2019; Wang et al., 2020). In particular, the disconnection hypothesis between different brain regions is considered to mainly contribute to cognitive decline in patients with SCD (Dillen et al., 2016). For instance, for the brain network based on resting-state functional magnetic resonance imaging (rs-fMRI), the identified connectivity disruption of SCD focused on the middle frontal gyrus, precuneus, and cingulate gyrus, which corresponded to the default mode network (DMN) (Greicius et al., 2004; Hafkemeijer et al., 2013; Xu et al., 2020b). Shu et al. analysed the graph theory metrics of structural brain network based on diffusion tensor imaging (DTI) and found that patients with SCD exhibited lower global efficiency and local efficiency of global graph metrics and reduced regional efficiency in the bilateral prefrontal regions and left thalamus (Shu et al., 2018). Moreover, the graph theoretic analysis of the topological properties of the morphological network based on structural magnetic resonance imaging (sMRI) showed that patients with SCD exhibiting lower network parameter values were associated with an increased risk of disease progression (Tijms et al., 2018). Therefore, these results demonstrated that patients with SCD have altered connectivity involving multimodal brain networks. In addition, recent studies have suggested that individuals with SCD exhibited volume atrophy and disconnection of the subcutaneous nuclei, such as basal forebrain, basal ganglia, and thalamus. Some researchers even proposed that the variation of the subcutaneous nuclei might be earlier than the cortex (Hampel et al., 2018; Scheef et al., 2019). However, most previous studies have focused on a single model of the brain network. The relationship between grey matter (GM) morphology, white matter structure and functional connectivity in SCD remains unclear.

Furthermore, to deal with the high-dimensional information yielded from multimodal brain networks, machine learning with multivariate pattern analysis was used to identify potential neuroimaging biomarkers and distinguish patients from HCs at an individual level. At the same time, it can reveal specific spatial distribution information useful exploring the brain network mechanisms underlying the cognitive impairment associated with AD. Previous studies, such as that by Yan et al. (2019) adopted a multimodal support vector machine (SVM) combined with structural and functional connectivity and achieved an accuracy of 98.58% in the AD group, 97.76% in the amnestic mild cognitive impairment (aMCI) group, and 80.24% in the SCD group. Compared with the single modal classification based on functional connectivity by Yu et al. (accuracy of 84.8% in AD), these results suggested that the integration of multimodal features can provide more comprehensive and insightful information than single modal features and achieve a better classification performance. However, to the best of our knowledge, there has been no study directly combining morphological, structural and functional brain networks to explore the relationship of different modalities and identify patients with SCD.

Given that individuals with SCD are often associated with abnormal multimodal brain network connectivity and the involvement of multiple brain regions, alongside the advantages of machine learning, we sought to apply multi-kernel SVM (MK-SVM) for the integration of morphological, structural and functional brain networks based on sMRI, DTI and fMRI. This study aimed to assess (a) whether specific altered patterns of network connectivity discovered by three modal brain networks can discriminate patients with SCD from HCs; (b) whether there is a correlation between different modal biomarkers and brain regions; and (c) whether the combination of multimodal network connectivity analyses may improve the accuracy of identifying patients with SCD from HCs.

## Materials and Methods

### Participants

The samples included in this study were acquired from the longitudinal follow-up data of China Longitudinal Aging Study (CLAS), a community-based study initiated in 2012. All individuals with Han Chinese nationality aged ≥60 years in Shanghai. A total of 67 right-handed participants involved in the present study, who underwent a screening process including medical history, epidemiological investigation, baseline and 7-year follow-up assessments of neuropsychological scale, and neuroimaging examinations. At baseline, the neuropsychological assessments included the Mini-Mental State Examination (MMSE) (Tombaugh and McIntyre, 1992), Montreal Cognitive Assessment (MoCA) (Nasreddine et al., 2005), Auditory Verbal Learning Test (AVLT) (Sheline et al., 1999), and Subjective Cognitive Decline Self-administered Questionnaire (SCD-9) (Shirooka et al., 2018). Meanwhile, T1-weighted MR imaging scan was performed. At 7-year follow-up, in addition to the neuropsychological scale mentioned above, multimodal MRI scans including T1WI, DTI and rs-fMRI were carried out. Therefore, research on the morphological, structural, and functional networks in this study was based on follow-up samples after 7 years. According to the follow-up results, 22 patients with SCD and 20 HCs were enrolled in our study. Due to the limitation of sample size, we considered the study classifies as pilot study.

The inclusion criteria for SCD were based on the conceptual framework proposed by the Subjective Cognitive Decline Initiative (SCD-I) (Jessen et al., 2014b), which included the following: (a) an onset age >60 years; (b) a self-perceived gradual decline in memory compared with a previous normal status within the last 5 years or as confirmed by a close caregiver; (c) MMSE and MoCA scores within the normal range; and (d) a Clinical Dementia Rating (CDR) score of 0. Those who did not experience any signs of cognitive decline and had neuropsychological tests scores in the normal range were included as HCs. The exclusion criteria of participants were as follows: (a) neurology-related or cerebral vascular diseases (e.g., Parkinson’s disease, brain tumours, or intracranial aneurysms); (b) systemic diseases that could cause cognitive impairments (e.g., thyroid dysfunctions, syphilis, HIV or severe anaemia); (c) severe schizophrenia according to their medical records; (d) severe problems in vision, hearing, or speaking; and (e) inability to participate actively in the neuropsychological evaluation.

This study was approved by the Institution’s Ethical Committee of Shanghai Mental Health Centre of Shanghai Jiao Tong University School of Medicine (NCT03672448). All participants provided written informed consent prior to any experimental procedures in the research. In terms of the statistical analysis of demographics and clinical characteristics between the SCD group and HC group, two-sample *t*-test or a chi-squared (χ2) test were performed by the Statistical Package for Social Science (SPSS, v20.0)^1^. The significance level was set at *P* < 0.05.

### Data Acquisition

T1-weighted structural imaging, DTI, and rs-fMRI scans were performed on each participant simultaneously. All MRI data were acquired on a 3.0 T MR scanner (Magnetom^®^ Verio; Siemens, Munich, Germany) using a 32-channel head coil. All participants were instructed to keep their eyes closed (but no fall asleep), think of nothing, and move as little as possible during the scan.

T1-weighted 3D high-resolution images were acquired using a magnetisation-prepared rapid gradient echo (MPRAGE) with the following parameters: repetition time (TR) = 2,300 ms, echo time (TE) = 2.98 ms, flip angle = 9 degrees, inversion time (TI) = 1,100 ms, matrix size = 240 × 256, field of view (FOV) = 240 × 256 mm^2^, slice number = 192, thickness = 1.2 mm and voxel size = 1.0 × 1.0 × 1.2 mm^3^. The scan lasted for 5 min and 12 s. DTI data were obtained using an echo planar imaging sequence with the following parameters: 64 non-collinear directions with a *b*-value = 1,000 s/mm^2^ and one additional image with no diffusion weighting (*b* = 0), TR = 13,700 ms, TE = 85 ms, FOV = 224 × 224 mm^2^, slice number = 75, thickness = 2 mm and voxel size = 2.0 × 2.0 × 2.0 mm^3^. In addition, the parameters of the rs-fMRI protocol were collected as follows: axial slices, TR = 2,000 ms, TE = 30 ms, flip angle = 90 degrees, FOV = 224 × 224 mm^2^, matrix size = 64 × 64, slice number = 31, thickness = 3.6 mm and voxel size = 3.5 × 3.5 × 3.6 mm^3^. Each scan collected 240 volumes with a scan time of 8 min and 6 s.

### Image Preprocessing

Brain tissue segmentation was performed using SPM12 (Ashburner and Ridgway, 2012). Individual T1-weighted 3D high-resolution images were segmented into the GM, white matter, and cerebrospinal fluid using a voxel-based morphometric analysis (Ashburner and Friston, 2000). The segmented GM images were realigned to the Montreal Neurologic Institute (MNI) space and normalised by DARTEL (Ashburner, 2007). Jacobian determinants were used to modulate and compensate for spatial normalisation effects (Mueller et al., 2019). A spatial smoothing process with a Gaussian kernel (full width at half maximum, 6 mm) was carried out to render the data more normally distributed and improve the signal-to-noise ratio (Shen and Sterr, 2013).

The PANDA toolbox (Cui et al., 2013) based on FSL (Jenkinson et al., 2012) was used for the pre-processing processes of DTI images, such as the removal of redundant scalp and brain tissues, and head motion and eddy current correction. In addition, the tensor model was fitted using a linear least-squares fitting method, and the fractional anisotropy (FA) value was calculated.

The processing of the fMRI scans was carried out by the Configurable Pipeline for the Analysis of Connectomes (C-PAC), which is a python-based pipeline tool making use of AFNI (Cox, 1996), ANTs (Tustison et al., 2014), FSL, and custom python code. Functional pre-processing included the following steps: (1) The first 10 time points were removed; (2) Slice-time correction was performed; (3) Images were de-obliqued; (4) Images were re-oriented into a right-to-left posterior-to-anterior inferior-to-superior orientation; (5) Motion correction was performed to averaged images to obtain motion parameters; (6) Skull stripping was performed; (7) The global mean intensity was normalised to 10,000; (8) Functional images were registered to anatomical space using a linear transformation, white-matter boundary-based transformation, and the prior white-matter tissue segmentation from FSL; (9) Motion artefacts were removed using ICA-based strategy for Automatic Removal of Motion Artefacts (ICA-AROMA) with partial component regression (Pruim et al., 2015); and (10) A nuisance signal regression was applied, including (a) mean values from the signal in the white matter and cerebrospinal fluid derived from the prior tissue segmentations transformed from anatomical to functional space, (b) motion parameters (six head-motion parameters, six head-motion parameters from one time point before, and the 12 corresponding squared items), (c) linear trends, and (d) global signal only for one set of strategies.

This entire analysis was accelerated and simplified through a cloud platform (^2^ Beijing Intelligent Brain Cloud, Inc.).

### Brain Network Construction

In present study, the Human Brainnetome (BN) Atlas (Fan et al., 2016) was used to divide the brain into 246 regions of interest (ROIs) to define the network nodes. Nevertheless, based on different modal brain networks, the definition of network edge was different.

#### Morphological Networks

Individual morphological brain networks were constructed by evaluating interregional similarity in the distribution of regional GM volume with the Kullback–Leibler divergence measure (Kong et al., 2014). First, the GM volume value of all voxels within the brain node were extracted. Second, the probability density function of these values was calculated with the kernel density estimation (KDE) (Wang et al., 2016). Next, the probability distribution function (PDF) was computed for the obtained probability density function. The variant KL divergence between any pair of ROI was calculated, resulting in a similarity matrix. KL divergence is a measure of the difference between two probability distributions from the perspective of probability theory, or of the information lost when one probability distribution approximates the other from the perspective of information theory. The following formula was used:

$$D_{K\,L}\,\left(P,Q\right)=\sum_{i=1}^{n}\left(P\,\left(i\right)\,l\,o\,g\,\frac{P\,\left(i\right)}{Q\,\left(i\right)}+Q\,\left(i\right)\,l\,o\,g\,\frac{Q\,\left(i\right)}{P\,\left(i\right)}\right)$$

where P and Q are two PDFs and n is the number of sample points. We selected *n* = 2^7^ in this study in reference to the research of Wang et al. (2016). Finally, a KL divergence-based similarity (KLS) measure were calculated to quantify morphological connectivity between two brain regions. The KLS was computed as below:

$$K\,L\,S\,\left(P,Q\right)=e^{-D_{K\,L}\,\left(P,Q\right)}$$

where *e* is a natural exponent. The KLS ranges from 0 to 1. The higher the value of KLS, the closer GM density distribution of the two brain regions is.

#### Structural Networks

After the pre-processing of DTI data, probabilistic tractography was used to construct the structural brain network (Behrens et al., 2007). For each seed region, probabilistic tractography was performed by seeding from all voxels of this region. For each voxel, 5,000 fibres were sampled. The connectivity probability from the seed region *i* to another region *j* was defined by the number of fibres passing through region *j* divided by the total number of fibres sampled from region *i* (5,000 × *n*, where n is the voxel number in region *i*). It is worth noting that the connection probability from *i* to *j* was not necessarily equal to that from *j* to *i*. These two probabilities were averaged to define the non-directional connection probability *Pij* between regions *i* and *j*.

#### Functional Networks

For the pre-processed fMRI data, the average time series of 246 ROIs was separately extracted to construct the functional brain network. The Pearson correlation coefficient of ROI pair-wise was defined as the edge of the functional connectivity, which resulted in 30,135 (246 × 245/2) edges.

The above structural and functional networks were accelerated and simplified through a cloud platform (see text footnote 2, Beijing Intelligent Brain Cloud, Inc.).

### Hubs of Each Imaging Modality

For each modal imaging (i.e., sMRI, DTI, and fMRI), the average value of the individual brain network was acquired to generate the group-average network. We identified the hub nodes by ranking the nodal degree. The rank 5% of brain regions were defined as the hubs of the brain network (Zhao et al., 2020).

### Feature Selection and Classification

In order to avoid the difficulty in identifying the contribution of kernel combination skills or feature selection to the final accuracy in the classification pipeline, we adopt the simplest method (*t*-test with *p* < 0.01) for feature selection. Meanwhile, the network-based statistic (NBS) (Zalesky et al., 2010) was used to conduct multiple comparisons correction for multimodal connections. The result of correction for multiple comparisons were listed in the Supplementary Figure 1. The LIBSVM toolbox^3^ for MATLAB was used to conduct the SVM classification (Xu et al., 2020a). Due to the limited samples, we used a leave one out cross-validation (LOOCV) strategy to evaluate the performance of the classification method. Specifically, inner cross-validation was carried out to determine the optimal parameter (hyper-parameter C for MK-SVM) and outer cross-validation was carried out to determine the classification performance. We compared the classification performance of single modes (i.e., sMRI, DTI and fMRI) and combinations of different modes (i.e., sMRI + DTI, fMRI + sMRI, fMRI + DTI, and fMRI + DTI + sMRI). Multi-kernel learning with a kernel combination trick was applied for multimodal information combination. The details of MK-SVM were listed as follows.

Assuming there are *n* training samples with connections values and graph metrics. ${\text{x}}_{\text{i}}^{\text{C}}$ and ${\text{x}}_{\text{i}}^{\text{G}}$y_i_ ∈ {1,−1} represent the connection value, the graph metrics, and its corresponding class label of the i-^th^ sample, respectively. MK-SVM solves the following primal problem:

$$\begin{array}{cc} \min_{\text{W}} & \frac{1}{2}\,\sum_{m=1}^{3}\beta_{m}\,{||w^{m}||}^{2}+C\,\sum_{i=1}^{\text{n}}\xi_{i}\\ s.t. & y_{i}\,\left(\sum_{m=1}^{3}\beta_{m}\,{\left(w^{m}\right)}^{T}\,\phi^{m}\,\left(x_{i}^{m}\right)+b\right)\geq 1-\xi_{i}\\ & \xi_{i}\geq 0,i=1,2,\ldots ,n \end{array}$$

where *ϕ^m^* represents mapping from the original space to the Represent Hilbert Kernel Space (RHKS), *w^m^* represents the normal vector of the hyperplane in RHKS, and β_*m*_ denotes the corresponding combining weight on the m-th modality. Next, the dual form of MK-SVM can be represented as:

$$\begin{array}{cc} \max_{\alpha } & \sum_{i=1}^{n}\alpha_{i}-\frac{1}{2}\,\sum_{i,j}\alpha_{i}\,\alpha_{j}\,y_{i}\,y_{j}\,\sum_{m=1}^{3}\beta_{m}\,k^{m}\,\left(x_{i}^{m},y_{i}^{m}\right)\\ s.t. & \sum_{\text{i}=1}^{\text{n}}\alpha_{i}\,y_{i}=0\\ & 0\leq \alpha_{\text{i}}\leq C,i=1,2,\ldots ,n \end{array}$$

where $k^{m}\,\left(x_{i}^{m},y_{i}^{m}\right)=\phi^{m}\,{\left(x_{i}^{m}\right)}^{T}\,\phi^{m}\,\left(x_{j}^{m}\right)$ is the kernel matrix onthe m-^th^ modality. After training the model, we tested the new samples *x* = {*x*_1_,*x*_2_,…,*x*_*M*_}. The kernel between the new test sample and the i-^th^ training sample on the m-^th^ modality is defined as $k^{m}\,\left(x_{i}^{m},x^{m}\right)=\phi^{m}\,{\left(x_{i}^{m}\right)}^{T}\,\phi^{m}\,\left(x^{m}\right)$. In the end, the predictive level based on MK-SVM can be formulated as follows:

$$f\,\left(x_{1},x_{2},\ldots ,x_{M}\right)=\text{sign}\,\left(\sum_{\text{i}=1}^{\text{n}}y_{i}\,\alpha_{i}\,\sum_{m=1}^{3}\beta_{m}\,k^{m}\,\left(x_{i}^{m},x^{m}\right)+b\right)$$

The proposed formulation of MK-SVM is similar, but different, to existing multi-kernel learning methods since β_*m*_ is selected based on the cross-validation scheme on the grid-searching space with constraints ∑_m_β_*m*_ = 1. The range of *C* was 2^–5^ to 2^5^.

### Consensus Connections

**FIGURE 1:**
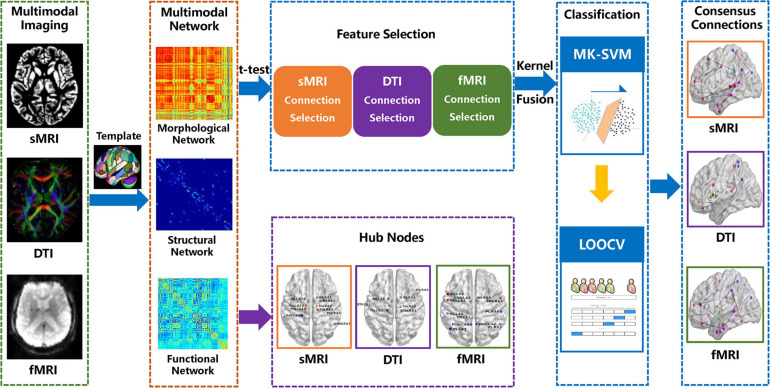
Procedures of data processing in the present study.

As mentioned above, we used the most commonly applied nested cross-validation scheme to evaluate the performance of the multi-kernel method proposed in this study. As the selected features by *t*-tests in each validation might be different, we record all the selected connection features during the training process. The consensus connections refer to the features that are consistently selected in all validations (Dosenbach et al., 2010; Zeng et al., 2012). In this study, we concentrate on consensus connections for each modal brain network. All data processing procedures in our study are shown in Figure 1.

### Robustness of Network Analysis

To demonstrate the robustness of the network analysis, we repeated the same network construction method and analysis process based on the automated anatomical labelling atlas (AAL) with 90 ROIs (Tzourio-Mazoyer et al., 2002).

## Results

### Demographic and Clinical Characteristics

The demographic and clinical characteristics of all participants are summarised in Table 1. The resultant scores of the SCD-9 in the SCD group were significantly higher than those in the HC group (*p* < 0.05). There were no significant differences with respect to age, education, sex, or any other scales.

**TABLE 1:** Demographics and clinical characteristics of patients with SCD and HC.

Characteristic/test	SCD	HC	T/χ^2^/Z	*P*
Age (years)	*74.0 ± 5.6*	71.8 ± 2.9	1.67^ a^	0.11
Education	10.1 ± 2.0	10.4 ± 3.0	0.00^ c^	1.00
Gender (F/M)	14/8	6/14	5.31^*b*^	0.05
MMSE	27.6 ± 1.8	28.2 ± 1.6	−1.13^ c^	0.26
MoCA	23.6 ± 3.9	24.1 ± 3.8	−0.48^ c^	0.63
AVLT-immediate recall	5.5 ± 1.9	4.8 ± 1.5	−0.98^*c*^	0.33
AVLT-short delayed recall	8.1 ± 2.6	8.2 ± 2.1	−0.14^*a*^	0.89
AVLT-long delayed recall	30.9 ± 7.7	33.2 ± 7.6	−1.00^*c*^	0.32
AVLT-recognition	10.2 ± 3.1	11.2 ± 3.0	−0.95^*c*^	0.34
SCD-9	3.8 ± 1.9	2.4 ± 2.0	0.58^ a^	0.03^∗^

*p < 0.05 indicates significant differences between the groups.

*^*a*^T value was obtained by using the two-sample *t*-test.*

*^*b*^χ^2^ value was obtained using the chi-square test.*

*^*c*^Z value obtained by using the rank-sum test.*

*MMSE, mini mental state examination; MoCA, Montreal Cognitive Assessment; AVLT, Auditory Verbal Learning Test; SCD-9, Subjective Cognitive Decline Self-administered Questionnaire. Data are presented as the mean ± standard deviation (SD). SCD, subjective cognitive decline; HC, healthy control.*

### Multimodal Brain Network Matrix

**FIGURE 2:**
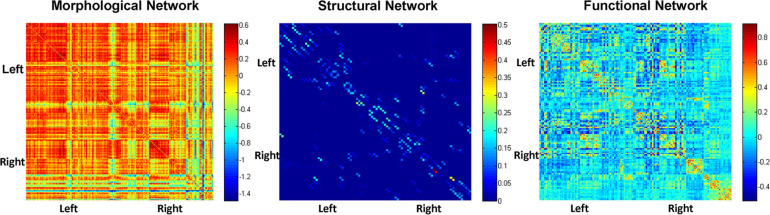
Adjacent matrices of HCs at the group level based on morphological, structural, and functional network.

Figure 2 depicts adjacent matrices of HCs at the group level based on morphological, structural, and functional network. The different colour reflects the weight value of the connectivity edges at group level. As shown in Figure 2, both functional and morphological networks, and particularly the functional networks, showed strong homotopic connections. As mentioned above, the network connectivity of different modalities pointed to different physiological mechanisms. The individual morphological brain networks in our study demonstrated that the morphological network showed strong contralateral homotopic connections, indicating that the GM density distributions in the same brain area on the left and right cerebral hemispheres were the most similar. Meanwhile, the weak homotopic connections between cortex and subcortex indicated that the GM densities of these two parts were quite different, resulting in lower morphological network connectivity. Thus, the mechanisms underlying the morphological network are basically consistent with the anatomical basis of the brain. Meanwhile, the structural network based on DTI exhibited sparse connections, and its connections were mainly short-distance fibre connections between the neighbouring areas. It corresponds to the pathway of white matter fibres in the structural brain network.

### Distribution of Hubs

**FIGURE 3:**
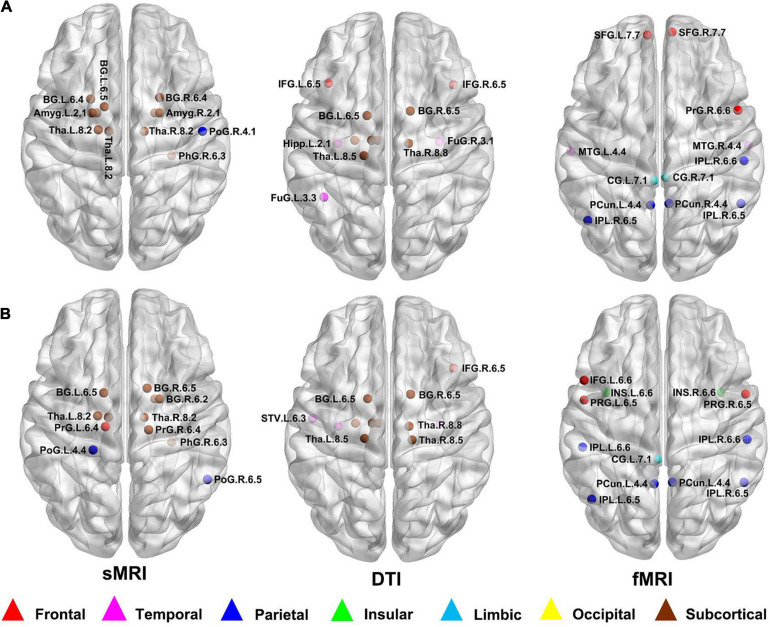
Hub nodes of the SCD and HC groups in different brain networks. **(A)** Hub nodes of SCD in morphological, structural and functional brain network based on sMRI, DTI and fMRI; **(B)** Hub nodes of HC in morphological, structural and functional brain network based on sMRI, DTI and fMRI. The hub nodes were mapped on the ICBM 152 template with the BrainNet Viewer package (http://nitrc.org/projects/bnv/). sMRI, structural magnetic resonance imaging; DTI, diffusion tensor imaging; fMRI, functional magnetic resonance imaging; SCD, subjective cognitive decline; HC, healthy control.

According to the definition of hub nodes in this study, the hub nodes of the SCD (Table 2) and HC groups (Table 3) based on three different modal networks were obtained. As shown in Figure 3, the distribution of hub nodes in the morphological and structural brain networks was similar, and most of them were located in the subcortical nuclei such as the hippocampus, thalamus, caudate nucleus, and amygdala. In contrast with the morphological and structural brain networks, the hub nodes of functional brain network were widely distributed in the frontal, temporal, and parietal lobes. Furthermore, by comparing the hub nodes between the SCD and HC groups in the same modal network, it was found that most of them overlapped. However, several specific hub nodes corresponded to the different groups. For instance, in the morphological network based on sMRI, the precentral gyrus (PrG) and the inferior parietal lobule (IPL) only appeared in the hub nodes of the HCs. In structural network based on DTI, the superior temporal gyrus (STG) only appeared in the hub node of the HC group, while the inferior frontal gyrus (IFG) only appeared in the SCD group as the hub node. Besides, in the functional brain network based on fMRI scans, the insula (INS) as one of the Hubs only appears in the HC group, while the middle temporal gyrus (MTG) as one of the Hubs only appears in the SCD group.

**TABLE 2:** Hubs of SCD based on different modal brain network.

sMRI		DTI		fMRI	
		
Label ID	ROI	Label ID	ROI	Label ID	ROI
156	PoG.R.4.1	107	FuG.L.3.3	64	PrG.R.6.6
226	BG.R.6.4	37	IFG.L.6.5	88	MTG.R.4.4
227	BG.L.6.5	38	IFG.R.6.5	144	IPL.R.6.5
225	BG.L.6.4	245	Tha.L.8.8	13	SFG.L.7.7
211	Amyg.L.2.1	227	BG.L.6.5	146	IPL.R.6.6
245	Tha.L.8.8	239	Tha.L.8.5	176	CG.R.7.1
114	PhG.R.6.3	246	Tha.R.8.8	87	MTG.L.4.4
212	Amyg.R.2.1	237	Tha.L.8.4	14	SFG.R.7.7
221	BG.L.6.2	228	BG.R.6.5	143	IPL.L.6.5
222	BG.R.6.2	215	Hipp.L.2.1	175	CG.L.7.1
234	Tha.R.8.2	103	FuG.L.3.1	154	PCun.R.4.4
233	Tha.L.8.2	104	FuG.R.3.1	153	PCun.L.4.4

*sMRI, structural magnetic resonance imaging; DTI, diffusion tensor imaging; fMRI, functional magnetic resonance imaging; SCD, subjective cognitive decline; ROI: region of interest.*

**TABLE 3:** The hubs of HC based on different modal brain network.

sMRI		DTI		fMRI	
		
Label ID	ROI	Label ID	ROI	Label ID	ROI
161	PoG.L.4.4	73	STG.L.6.3	174	INS.L.6.6
144	IPL.R.6.5	245	Tha.L.8.8	145	IPL.L.6.6
228	BG.R.6.5	240	Tha.R.8.5	39	IFG.L.6.6
245	Tha.L.8.8	38	IFG.R.6.5	144	IPL.R.6.5
114	PhG.R.6.3	246	Tha.R.8.8	61	PrG.L.6.5
60	PrG.R.6.4	227	BG.L.6.5	146	IPL.R.6.6
227	BG.L.6.5	237	Tha.L.8.4	174	INS.R.6.6
212	Amyg.R.2.1	239	Tha.L.8.5	175	CG.L.7.1
59	PrG.L.6.4	228	BG.R.6.5	143	IPL.L.6.5
222	BG.R.6.2	215	Hipp.L.2.1	62	PrG.R.6.5
234	Tha.R.8.2	103	FuG.L.3.1	154	PCun.R.4.4
233	Tha.L.8.2	104	FuG.R.3.1	153	PCun.L.4.4

*sMRI, structural magnetic resonance imaging; DTI, diffusion tensor imaging; fMRI, functional magnetic resonance imaging; HC, healthy control; ROI, region of interest.*

### Classification

**FIGURE 4:**
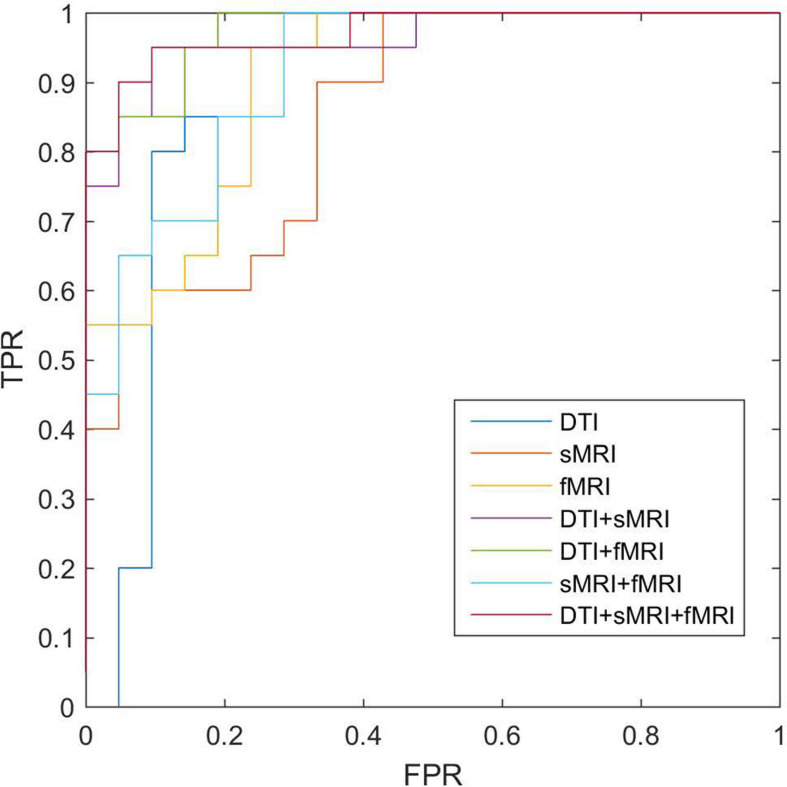
ROC of classifications based on different modalities. sMRI, structural magnetic resonance imaging; DTI, diffusion tensor imaging; fMRI, functional magnetic resonance imaging; ROC, receiver operating characteristic.

After feature selection of the morphological, structural, and functional network connections by *t*-tests, MK-SVM was applied to combine the selected multimodal connections to identify individuals with SCD from HCs. As shown in Table 4 and Figure 4, for the single modality, the classification accuracy of the morphological, structural and functional networks was 73.17, 80.49, and 85.37%, respectively. That is, the functional network constructed by fMRI exhibited the highest accuracy rate, followed by the structural network constructed by DTI; finally, the morphological network constructed by GM volume showed the lowest accuracy rate. Furthermore, combining the morphological and structural connections (sMRI + DTI), functional and morphological connections (fMRI + sMRI), and functional and structural connections (fMRI + DTI), the accuracy of classification increased to 85.37, 87.80, and 90.24%, respectively. In particular, the best classification performance was obtained by combining the selected connections of three modalities, with an accuracy of 92.68%, sensitivity of 95.00% and specificity of 90.48%. These results suggested that the combination of multimodal network features could significantly improve the classification performance.

**TABLE 4:** Classification performance of different modalities.

Modalities	Accuracy (%)	Specificity (%)	Sensitivity (%)	AUC
sMRI	73.17	80.00	66.67	0.8785
DTI	80.49	85.00	76.19	0.8523
fMRI	85.37	90.00	80.95	0.9047
sMRI + DTI	85.37	95.00	76.19	0.9142
fMRI + sMRI	87.80	95.00	80.95	0.9714
fMRI + DTI	90.24	90.00	80.95	0.9619
fMRI + DTI + sMRI	92.68	95.00	90.48	0.9738

*AUC, area under the curve; sMRI, structural magnetic resonance imaging; DTI, diffusion tensor imaging; fMRI, functional magnetic resonance imaging.*

### Consensus Connections

**FIGURE 5:**
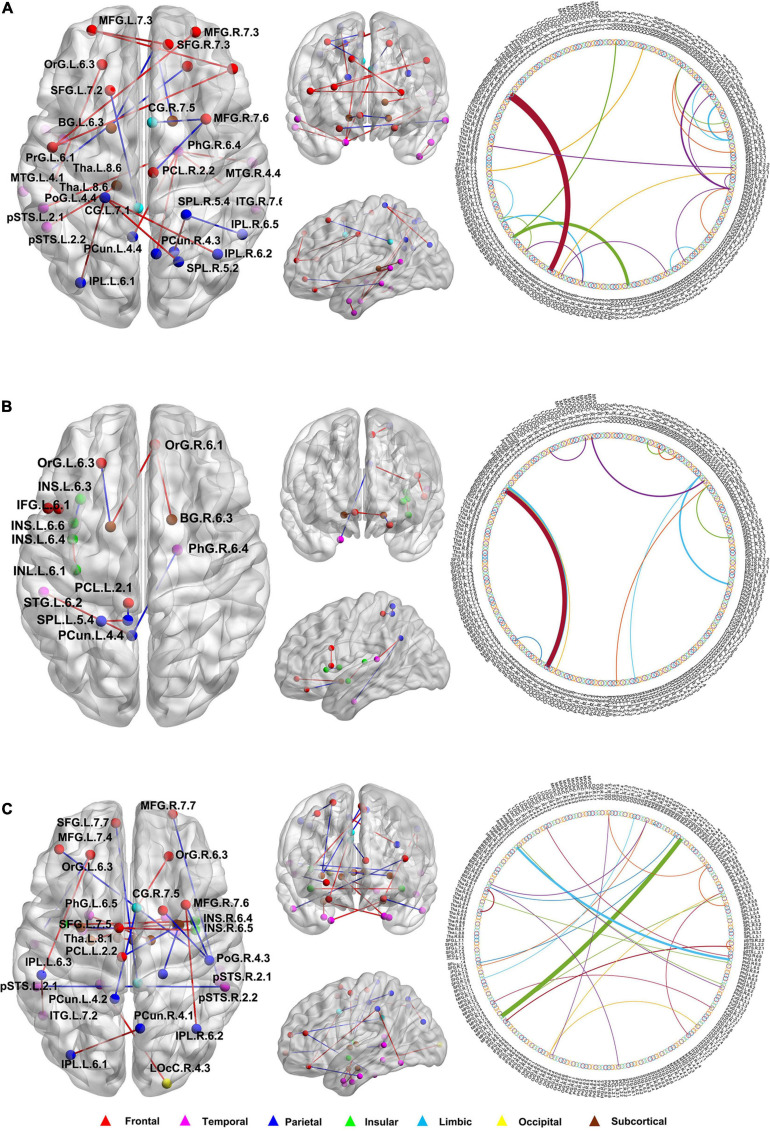
The distribution of consensus connections identified by different modalities. **(A)** Morphological brain network based on sMRI; **(B)** Structural brain network based on DTI; **(C)** Functional brain network based on fMRI. The consensus connections were mapped on the ICBM 152 template with the BrainNet Viewer package (http://nitrc.org/projects/bnv/). Red and blue lines represent the increased and decreased connectivity weight of the SCD group, respectively. sMRI, structural magnetic resonance imaging; DTI, diffusion tensor imaging; fMRI, functional magnetic resonance imaging.

In this study, we further identified the consensus connections for each modal brain network (Figure 5). The morphological brain network based on sMRI yielded a total of 23 consensus connections (Table 5), including 7 positive connections and 16 negative connections, which were mainly associated with the frontal lobe (orbital gyrus [OrG], middle frontal gyrus [MFG], superior frontal gyrus [SFG]), temporal lobe (MTG, entorhinal cortex [EC]), parietal lobe (inferior parietal lobule [IPL]), and subcortical nuclei (nucleus accumbens [NAC], occipital thalamus [Otha]). Meanwhile, the structural brain network based on DTI had a total of 12 consensus connections (Table 6), including 7 positive connections and 5 negative connections, which were mainly distributed in the parietal lobe (precuneus [Pcun]), frontal lobe (OrG), insula (INS), temporal lobe (EC), and subcortical nuclei (NAC). In addition, the functional brain network based on fMRI scans had a total of 24 consensus connections (Table 7), which were mainly distributed in the parahippocampal gyrus (PhG), INS, SFG, IPL, and subcortical nuclei (medial pre-frontal thalamus [mPFtha], pre-motor thalamus[mPMtha], rostral temporal thalamus [rTtha], dorsolateral putamen [dlPu], lateral amygdala[lAmyg]). Eleven of these connections were positive connections, suggesting that the strength of functional connections of patients with SCD was stronger than that of HCs, and mainly distributed in the cortical-cortical connections between the frontal lobe (MFG, SFG) and the temporal lobe (posterior Superior Temporal Sulcus [pSTS], inferior temporal gyrus [ITG]) and parietal lobe (postcentral gyrus [PoG]). The other 13 negative connections were mainly distributed in the cortical-subcortical connections between the temporal lobe (PhG) and the subcortical nuclei (Tha, amygdala [Amyg]). Therefore, our results indicated that the consensus connections of these three modal networks were involved in a wide range of cortical-subcortical circuits, especially the connection between the cortex and the subcutaneous nucleus including the thalamus, basal ganglia, and amygdala. Furthermore, there existed both positive and negative consensus connections across the three modalities. Positive connections were mainly distributed in the frontal lobe-related connections, and negative connections were mainly distributed in the temporal lobe and subcortical nuclei-related connections.

**TABLE 5:** Consensus connections identified by the morphological brain network based on sMRI.

ROI	ROI	Mean value		*P*-value
			
		SCD	HC	
OrG.R.6.1	BG.L.6.3	0.296	0.241	7.34 × 10^−5^
OrG.R.6.1	BG.R.6.3	0.480	0.256	8.40 × 10^−5^
MFG.R.7.6	PCL.R.2.2	0.767	0.509	1.27 × 10^−4^
PhG.R.6.4	PCun.L.4.4	0.094	0.273	2.91 × 10^−4^
SPL.R.5.4	IPL.R.6.5	0.387	0.027	3.19 × 10^−4^
MFG.R.7.3	PrG.L.6.1	0.461	0.512	4.47 × 10^−4^
MFG.R.7.6	CG.R.7.5	0.115	0.002	5.64 × 10^−4^
SFG.L.7.2	CG.L.7.1	0.342	0.257	7.98 × 10^−4^
pSTS.L.2.2	Tha.L.8.6	0.075	0.297	8.36 × 10^−4^
IFG.R.6.2	PrG.L.6.1	0.046	0.452	1.15 × 10^−3^
MTG.L.4.2	ITG.L.7.3	0.540	0.634	1.15 × 10^−3^
SPL.R.5.4	PCun.R.4.3	0.428	0.446	1.36 × 10^−3^
PhG.R.6.4	PCun.R.4.1	0.169	0.225	1.39 × 10^−3^
OrG.L.6.3	pSTS.L.2.1	0.118	0.602	1.58 × 10^−3^
PhG.R.6.4	pSTS.L.2.1	0.146	0.367	1.67 × 10^−3^
SFG.R.7.3	MFG.L.7.3	0.466	0.609	1.72 × 10^−3^
SPL.R.5.2	PoG.L.4.4	0.208	0.673	1.76 × 10^−3^
OrG.R.6.3	MTG.L.4.1	0.019	0.004	1.92 × 10^−3^
IPL.L.6.1	PoG.L.4.4	0.002	0.343	1.95 × 10^−3^
MTG.R.4.4	PhG.R.6.4	0.011	0.253	1.99 × 10^−3^
MFG.L.7.3	IFG.R.6.2	0.278	0.483	2.25 × 10^−3^
IPL.R.6.2	PoG.L.4.4	0.438	0.777	2.29 × 10^−3^
ITG.R.7.6	PhG.R.6.4	0.197	0.232	2.57 × 10^−3^

*sMRI, structural magnetic resonance imaging; SCD, subjective cognitive decline; HC, healthy control; ROI, region of interest.*

**TABLE 6:** Consensus connections identified by structural brain network based on DTI.

ROI	ROI	Mean value		*P*-value
			
		SCD	HC	
BG.L.6.3	OrG.R.6.1	0.296	0.241	7.34 × 10^−5^
BG.R.6.3	OrG.R.6.1	0.480	0.256	8.40 × 10^−5^
PCun.L.4.4	PhG.R.6.4	0.094	0.273	2.91 × 10^−4^
PCun.L.4.3	MVOcC .L.5.2	0.002	0.003	3.59 × 10^−4^
MVOcC .L.5.3	LOcC.L.4.4	0.021	0.019	8.17 × 10^−4^
INS.L.6.1	INS.L.6.4	0.104	0.072	8.47 × 10^−4^
INS.L.6.6	INS.L.6.3	0.116	0.180	9.02 × 10^−4^
SPL.L.5.4	PCun.L.4.2	0.055	0.052	9.25 × 10^−4^
PCL.L.2.1	PCun.L.4.2	0.216	0.242	1.70 × 10^−3^
PCun.L.4.4	STG.L.6.2	0.003	0.001	2.22 × 10^−3^
IFG.L.6.1	IFG.L.6.6	0.086	0.047	2.32 × 10^−3^
OrG.L.6.3	BG.L.6.3	0.002	0.005	2.42 × 10^−3^

*DTI, diffusion tensor imaging; SCD, subjective cognitive decline; HC, healthy control; ROI, region of interest.*

**TABLE 7:** Consensus connections identified by functional brain network based on fMRI.

ROI	ROI	Mean value		*P*-value
			
		SCD	HC	
MFG.R.7.6	PoG.R.4.3	0.221	0.204	4.19 × 10^−5^
PhG.R.6.5	Hipp.L.2.1	0.095	0.557	8.91 × 10^−5^
Tha.L.8.1	Tha.R.8.4	0.700	0.812	3.22 × 10^−4^
MFG.R.7.7	pSTS.R.2.1	0.154	0.027	3.60 × 10^−4^
pSTS.L.2.1	pSTS.R.2.2	0.321	0.316	7.94 × 10^−4^
INS.R.6.5	BG.L.6.6	0.292	0.075	8.47 × 10^−4^
IPL.L.6.1	PCun.R.4.1	0.639	0.660	8.66 × 10^−4^
PhG.L.6.1	Amyg.R.2.2	0.143	0.377	1.39 × 10^−3^
OrG.L.6.3	ITG.L.7.4	0.443	0.156	1.40 × 10^−3^
MFG.R.7.6	IPL.R.6.2	0.540	0.853	1.41 × 10^−3^
PCL.L.2.2	BG.R.6.6	0.416	0.097	1.44 × 10^−3^
PhG.L.6.6	LOcC .R.4.3	0.109	0.425	1.79 × 10^−3^
SFG.R.7.4	PoG.R.4.3	0.282	0.197	1.90 × 10^−3^
PCun.L.4.2	CG.R.7.5	0.244	0.059	2.00 × 10^−3^
SFG.L.7.5	INS.R.6.5	0.154	0.282	2.10 × 10^−3^
PhG.R.6.1	Amyg.R.2.2	0.200	0.577	2.12 × 10^−3^
PoG.R.4.4	BG.R.6.6	0.384	0.075	2.14 × 10^−3^
PhG.L.6.5	Tha.R.8.2	0.138	0.232	2.27 × 10^−3^
ITG.L.7.2	IPL.L.6.3	0.105	0.690	2.35 × 10^−3^
INS.L.6.4	INS.R.6.4	0.516	0.587	2.38 × 10^−3^
OrG.L.6.3	IPL.L.6.3	0.173	0.192	2.40 × 10^−3^
MFG.L.7.4	ITG.R.7.7	0.167	0.140	2.49 × 10^−3^
SFG.L.7.7	CG.R.7.1	0.749	0.360	2.51 × 10^−3^
OrG.R.6.3	CG.R.7.5	0.170	0.217	2.57 × 10^−3^

*fMRI, functional magnetic resonance imaging; SCD, subjective cognitive decline; HC, healthy control; ROI, region of interest.*

### Robustness of Network Analysis

As mentioned above, we repeated the same network construction method and analysis process based on the AAL, with 90 ROIs, to demonstrate the robustness of our results. Supplementary Figure 2 describes the multimodal networks at the group level. In terms of the distribution of hub nodes and consensus connections, the AAL90 template and BN template partially overlapped, involving the cortical-subcortical brain regions and their connections (Supplementary Figures 3, 4). However, it is worth noting that the number of these features based on the AAL template was significantly reduced compared with the BN template, especially in subcortical nuclei, such as the thalamus. This may be because the AAL template has not yet subdivided the subcortical nuclei into more detailed subregions, resulting in a significant reduction in the number of subcortical nuclei distribution. In addition, the classification results based on the AAL template also demonstrated that compared with the classification accuracy of single modality of the morphological, structural, and functional network (73.17, 58.54, and 78.05%, respectively), the combination of three modalities could significantly improve the classification accuracy of SCD (87.80%) (Supplementary Table 1 and Supplementary Figure 5).

## Discussion

In this study, we constructed the morphological, structural, and functional brain networks based on sMRI, DTI and fMRI, respectively, and aimed to explore the biomarkers of brain network in individuals with SCD. Our results indicated that the combination of three modalities using MK-SVM could significantly improve the classification performance of individuals with SCD. More importantly, the consensus connections based on the morphological, structural, and functional networks identified in our study highlight the role of the cortical-subcortical circuit in the pathological mechanisms associated with individuals with SCD.

### Alterations in Morphological Brain Network

In our study, individual morphological brain network was constructed based on the KLS method (Kong et al., 2014). Compared with the previous group-level morphological brain network obtained by estimating the interregional correlations of morphological features (e.g., cortical thickness, cortical surface area or GM volume) (He et al., 2008; Evans, 2013; Matsuda, 2016), the KLS-based morphological brain network could generate an individualised brain network for each participant according to customised brain network nodes defined by specific brain atlases (Kong et al., 2014). Therefore, it is more suitable to construct efficient and stable morphological brain networks and depict complex topological properties of brain network. For the distribution of hubs and consensus connections of morphological brain networks, our results indicated that most of them were involved in cortical-subcortical circuits. Furthermore, the consensus connections among the temporal lobe, parietal lobe, and subcortical nuclei of individuals with SCD were weaker than those of HCs. According to the definition of KLS, the lower value of the KLS, the greater the difference between two brain regions in GM volume distributions (Wang et al., 2016). Our results hinted the heterogeneity of GM volume variation of cortex and subcortical nuclei in SCD patients. Although previous studies have also demonstrated that individuals with SCD exhibited decreased GM volume in hippocampus, entorhinal cortex and amygdala compared to the HCs (Jessen et al., 2006; Stewart et al., 2011; Niemantsverdriet et al., 2018), our results further highlighted the differences in volume changes of brain regions distributed in the cortex and subcortical nuclei.

### Alterations in Structural Brain Network

In terms of structural networks based on DTI, most previous structural networks were constructed using deterministic fibre tracking algorithms (Shu et al., 2018; Yan et al., 2018). In comparison, the probabilistic fibre tracking algorithm of this study considered the uncertainty of fibre direction estimation, thereby improving the accuracy of white matter fibre tracking (Ratnarajah et al., 2012). Regarding the distribution of hubs and consensus connections of structural network, our results indicated that the discriminative features of structural network based on DTI and the morphological network based on sMRI involved in overlapped and multiple cortical-subcortical brain regions, such as the frontal lobe, PhG, Tha, and BG. It is consistent with the anatomical basis of brain’s GM and white matter distributed. Structural connections reflected the degree of projection connections of white matter fibres between different brain regions (Morris et al., 2008), our findings revealed that SCD patients presented abnormalities of multiple white matter fibre bundles in cortical-subcortical circuit. Previous studies have indicated that most cholinergic fibres originate from the projection of cholinergic neurons in the subcortical nuclei, which connected with hippocampus complex and the cortex through thalamus to constitute the basal forebrain-thalamus-cortex circuit (Hanakawa et al., 2017; Meng et al., 2018; Villagrasa et al., 2018). The input and output of pathway, such as Papez circuit, have been demonstrated to play an important role in memory, learning and attention (Semba, 2000; Aggleton et al., 2016; Agostinelli et al., 2019).

### Alterations in Functional Brain Network

Regarding the functional brain networks based on rs-fMRI, functional connections were quantified by calculating the pairwise Pearson’s correlation coefficients of blood oxygen level dependent (BOLD) time series obtained for each ROI (Raichle et al., 2001). Based on the distribution of hubs in SCD, we found that most of them, such as the SFG, MTG, cingulate gyrus, and precuneus, were located in the DMN. Similar to previous studies (Greicius et al., 2004; Wang et al., 2013; Chiesa et al., 2019), our study also demonstrated the important role of DMN in the functional brain networks of individuals with SCD. In addition, it is worth noting that compared with the morphological and structural networks, the functional networks exhibited a larger number and wider range of consensus connections between the cortex and subcortical brain regions. Furthermore, we found that the decreased functional connections were mainly distributed in the temporal lobe, thalamus and insula, which might lead to memory impairments (Aggleton et al., 2016). Meanwhile, the increased functional connections related to the frontal lobe might be attributed to the compensatory changes in the functional brain network in the transition stage of SCD.

### The Relationship Between the Modalities

Based on the alterations of three different modalities mentioned above, we found that there exists correlation between the morphological, structural, and functional brain networks. Regarding morphological and the structural networks, our results indicated that the hubs and discriminative consensus connections of structural network based on DTI and the morphological network based on sMRI involved in overlapped brain regions, such as the frontal lobe, PhG, Tha, and BG. It is consistent with the anatomical basis of grey matter and white matter distribution in the cortical-subcortical circuit. The related brain regions (e.g., hippocampus, parahippocampal gyrus, cingulate gyrus, amygdala, entorhinal cortex, basal ganglia, and thalamus) were anatomically connected by the white matter fibre bundles such as the fornix, corpus callosum, and external capsule (Schmahmann et al., 2008). Therefore, the alterations between these two modalities were similar. Moreover, compared with the morphological and structural networks, the functional networks exhibited a larger number and wider range of consensus connections between the cortex and subcortical nuclei. As Honey et al. (2009) have demonstrated in previous research, functional connectivity was frequently found between regions without direct structural linkage; nevertheless, its strength and spatial statistical values remained constrained by the large-scale anatomical structure of the brain and reflected the underlying pathologic alterations. Therefore, different modal brain networks can provide complementary information for detecting abnormalities in SCD individuals.

### Abnormalities in the Cortical-Subcortical Circuit

Hub nodes play a critical role in global information transfer and seem to be vulnerable and preferentially affected in patients with AD (Dai and He, 2014). Our results found the disappearance of some hubs in the SCD group, which suggested the brain network integration function of SCD patients may have changed. The reason may be related to the early pathological changes of AD. In additon, for the distribution of the hubs and consensus connections of the morphological, structural, and functional brain networks, our results point to significant abnormalities in the cortical-subcortical brain regions and the connections between them in SCD. In particular, the connections between the subcutaneous nucleus (e.g., BG, amygdala, and thalamus) and the limbic system (e.g., hippocampus, parahippocampal gyrus, cingulate gyrus and entorhinal cortex) and cortex, corresponding to the cortical-subcortical circuit, was significantly aberrant in SCD. Among them, all the subcortical nucleus we identified are highly complex of subnuclei. For instance, thalamus includes more than 10 subnuclei with distinct connections. And the basal nuclei and thalamus participate in many different neuronal pathways, such as cholinergic pathways, with functions related to memory, learning, emotion, attention (Ballinger et al., 2016). Previous studies have found the fewer cholinergic neurons and abnormal amyloid-beta accumulation in cholinergic pathways, are considered important factors leading to the decline of cognitive function in AD (Saxena and Caroni, 2011; Baker-Nigh et al., 2015; Fernandez-Cabello et al., 2020). Therefore, our study provided important clues for the early identification and mechanisms exploration of SCD.

### Classification of MK-SVM

In addition, after feature selection of the morphological, structural, and functional network connections by a *t*-test, MK-SVM was applied to combine these features for the classification. For the single modality, we found that the functional network based on fMRI has the highest accuracy rate compared to the morphological and structural networks. This is consistent with the previous study by Yan et al. (2019) that focused on SCD classification based on structural and functional networks. Thus, we speculated that in this stage of SCD, functional changes in the brain were more significant than structural changes in the GM and white matter. In addition, combining two multimodal modalities improved the classification accuracy. Furthermore, the combination of three modalities achieved the best classification performance. The MK-SVM in our study, as an innovative and optimised multimodal information fusion method, can adaptively learn the optimal combined core from a set of base cores and solve the problem of kernel functions selection. Meanwhile, it may address the imbalanced dimension issue across different modalities to some extent and partially alleviate the high-dimensional curve representing multiple features to discriminate individuals with SCD from HCs. Compared with certain previously published research (Yan et al., 2019; Chen et al., 2020), we obtained a better classification performance in response to a multimodal brain network combination. Combined with the model validation based on the AAL template, our findings emphasised that the combination of multimodal brain networks may be considered a potential approach for the early discrimination of individuals with SCD from HCs.

### Limitations and Future Directions

Although our study sought to establish a new perspective to explore the brain network mechanisms associated with SCD and early-stage AD identification, several limitations exist with scope for further study. Firstly, a large sample size and multi-centre data are essential to training and validating models. The participant numbers are small for a multi-variate approach. Therefore, the study classifies as pilot study. Although our research has confirmed the stability and repeatability of the methodology based on the AAL template. In future work, we need to use large samples and multi-centre data to further verify the robustness of our proposed method and the repeatability of the results. Secondly, a follow-up study should be carried out for different stages of AD using longitudinal data. In this study, we only detected brain network abnormalities and performed the individual identification in individuals with SCD; longitudinal follow-up studies of the different stages of AD are needed to identify the early and specific imaging markers for diagnosis and prediction. Thirdly, a combination of multimodal diagnostic information should be carried out. We only used different modal brain network connections for the classification of SCD. In the future, we may attempt to identify and explore the pathological mechanisms associated with SCD by combining multimodal diagnostic information such as that stemming from Positron Emission Tomography (PET), Electroencephalography (EEG), and blood biomarker information.

## Conclusion

We applied the morphological, structural, and functional brain networks based on sMRI, DTI, and fMRI to investigate the pathological mechanisms and potential biomarkers of individuals with SCD. The discriminative connections of three modal brain networks shed light on the abnormality of cortical-subcortical circuit in SCD. Furthermore, the disconnection between different brain regions might lead to the cognitive decline in patients with SCD. In addition, the combination of three modalities with MK-SVM achieved the best classification performance for SCD. Our findings provided novel insights into the pathological mechanisms associated with patients with SCD presenting with early AD pathologies, which will thereby contribute to the development of more effective diagnostic tools and therapies for preclinical stages of AD.

## Data Availability Statement

The original contributions presented in the study are included in the article/Supplementary Material, further inquiries can be directed to the corresponding authors.

## Ethics Statement

The studies involving human participants were reviewed and approved by the Institution’s Ethical Committee of Shanghai Mental Health Center of Shanghai Jiao Tong University School of Medicine. The patients/participants provided their written informed consent to participate in this study.

## Author Contributions

XX and WL designed the study and drafted the manuscript. MZ collected the MRI data. LY and TW diagnose the subjects. XX, BX, and HL analyzed and interpreted the results of the data. SX and PW revised the manuscript. All authors approved the final manuscript.

## Conflict of Interest

HL and BX were employed by the company Beijing Intelligent Brain Cloud Inc., Beijing, China. The remaining authors declare that the research was conducted in the absence of any commercial or financial relationships that could be construed as a potential conflict of interest.

## Footnotes

1 http://www.spss.com/

2 http://www.humanbrain.cn

3 https://www.csie.ntu.edu.tw/∽cjlin/libsvm/
