# Supplementary material for: Morphological, Structural, and Functional Networks Highlight the Role of the Cortical-Subcortical Circuit in Individuals With Subjective Cognitive Decline
Source: Front Aging Neurosci. 2021 Jul 9;13:688113. doi: 10.3389/fnagi.2021.688113 (PMC8299728; doi:10.3389/fnagi.2021.688113)

**TABLE S1** TClassification performance of different modalities based on the AAL 90 template.

| Modalities | Accuracy (%) | Specificity  (%) | Sensitivity  (%) | AUC |
| --- | --- | --- | --- | --- |
| sMRI | 73.17 | 75.00 | 71.43 | 0.76 |
| DTI | 58.54 | 55.00 | 61.90 | 0.59 |
| fMRI | 78.05 | 75.00 | 80.95 | 0.77 |
| sMRI+ DTI | 78.05 | 75.00 | 80.95 | 0.85 |
| fMRI+sMRI | 85.37 | 80.00 | 90.48 | 0.91 |
| fMRI+DTI | 82.93 | 60.00 | 76.19 | 0.87 |
| fMRI+DTI+sMRI | 87.80 | 85.00 | 90.48 | 0.94 |

AAL, automated anatomical labelling atlas

**TABLE S2** Brain areas and their abbreviations in the AAL 90 template.

| Regions | Abb. | Regions | Abb. |
| --- | --- | --- | --- |
| Precental gyrus | PreCG | Lingual gyrus | LING |
| Superior frontal gyrus, dorsolateral | SFGdor | Superior occipital gyrus | SOG |
| Superior frontal gyrus, orbital part | ORBsup | Middle occipital gyrus | MOG |
| Middle frontal gyrus | MFG | Inferior occipital gyrus | IOG |
| Middle frontal gyrus, orbital part | ORBmid | Fusiform gyrus | FFG |
| Inferior frontal gyrus, opercular part | IFGoperc | Postcentral gyrus | PoCG |
| Inferior frontal gyrus, triangular part | IFGtriang | Superior parietal gyrus | SPG |
| Inferior frontal gyrus, orbital part | ORBinf | Inferior parietal, but supramarginal and angular gyri | IPL |
| Rolandic operculum | ROL | Supramarginal gyrus | SMG |
| Supplementary motor area | SMA | Angular gyrus | ANG |
| Olfactory cortex | OLF | Precuneus | PCUN |
| Superior frontal gyrus, medial | SFGmed | Paracentral lobule | PCL |
| Superior frontal gyrus, medial orbital | ORBsupmd | Caudate nucleus | CAU |
| Gyrus rectus | REC | Lenticular nucleus putamen | PUT |
| Insula | INS | Lenticular nucleus, pallidum | PAL |
| Anterior cingulate and paracingulate gyri | ACG | Thalamus | THA |
| Median cingulate and paracingulate gyri | MCG | Heschl gyrus | HES |
| Posterior cingulate gyrus | PCG | Superior temporal gyrus | STG |
| Hippocampus | HIP | Temporal pole: superior temporal gyrus | TPOsup |
| Parahippocampal gyrus | PHG | Middle temporal gyrus | MTG |
| Amygdala | AMYG | Temporal pole: middle temporal gyrus | TPOmid |
| Calcarine ﬁssure and surrounding cortex | CAL | Inferior temporal gyrus | ITG |
| Cuneus | CUN |  |  |

**TABLE S3** Brain areas and their abbreviations in the Human Brainnetome template.

| Lobe | Gyrus | Left and Right Hemisphere | Anatomical and modified Cyto-architectonic descriptions |
| --- | --- | --- | --- |
| **Frontal Lobe** | SFG, Superior Frontal Gyrus | SFG_L(R)_7_1 | A8m, medial area 8 |
|  |  | SFG_L(R)_7_2 | A8dl, dorsolateral area 8 |
|  |  | SFG_L(R)_7_3 | A9l, lateral area 9 |
|  |  | SFG_L(R)_7_4 | A6dl, dorsolateral area 6 |
|  |  | SFG_L(R)_7_5 | A6m, medial area 6 |
|  |  | SFG_L(R)_7_6 | A9m,medial area 9 |
|  |  | SFG_L(R)_7_7 | A10m, medial area 10 |
|  | MFG, Middle Frontal Gyrus | MFG_L(R)_7_1 | A9/46d, dorsal area 9/46 |
|  |  | MFG_L(R)_7_2 | IFJ, inferior frontal junction |
|  |  | MFG_L(R)_7_3 | A46, area 46 |
|  |  | MFG_L(R)_7_4 | A9/46v, ventral area 9/46 |
|  |  | MFG_L(R)_7_5 | A8vl, ventrolateral area 8 |
|  |  | MFG_L(R)_7_6 | A6vl, ventrolateral area 6 |
|  |  | MFG_L(R)_7_7 | A10l, lateral area10 |
|  | IFG, Inferior Frontal Gyrus | IFG_L(R)_6_1 | A44d,dorsal area 44 |
|  |  | IFG_L(R)_6_2 | IFS, inferior frontal sulcus |
|  |  | IFG_L(R)_6_3 | A45c, caudal area 45 |
|  |  | IFG_L(R)_6_4 | A45r, rostral area 45 |
|  |  | IFG_L(R)_6_5 | A44op, opercular area 44 |
|  |  | IFG_L(R)_6_6 | A44v, ventral area 44 |
|  | OrG, Orbital Gyrus | OrG_L(R)_6_1 | A14m, medial area 14 |
|  |  | OrG_L(R)_6_2 | A12/47o, orbital area 12/47 |
|  |  | OrG_L(R)_6_3 | A11l, lateral area 11 |
|  |  | OrG_L(R)_6_4 | A11m, medial area 11 |
|  |  | OrG_L(R)_6_5 | A13, area 13 |
|  |  | OrG_L(R)_6_6 | A12/47l, lateral area 12/47 |
|  | PrG, Precentral Gyrus | PrG_L(R)_6_1 | A4hf, area 4(head and face region) |
|  |  | PrG_L(R)_6_2 | A6cdl, caudal dorsolateral area 6 |
|  |  | PrG_L(R)_6_3 | A4ul, area 4(upper limb region) |
|  |  | PrG_L(R)_6_4 | A4t, area 4(trunk region) |
|  |  | PrG_L(R)_6_5 | A4tl, area 4(tongue and larynx region) |
|  |  | PrG_L(R)_6_6 | A6cvl, caudal ventrolateral area 6 |
|  | PCL, Paracentral Lobule | PCL_L(R)_2_1 | A1/2/3ll, area1/2/3 (lower limb region) |
|  |  | PCL_L(R)_2_2 | A4ll, area 4, (lower limb region) |
| **Temporal Lobe** | STG, Superior Temporal Gyrus | STG_L(R)_6_1 | A38m, medial area 38 |
|  |  | STG_L(R)_6_2 | A41/42, area 41/42 |
|  |  | STG_L(R)_6_3 | TE1.0 and TE1.2 |
|  |  | STG_L(R)_6_4 | A22c, caudal area 22 |
|  |  | STG_L(R)_6_5 | A38l, lateral area 38 |
|  |  | STG_L(R)_6_6 | A22r, rostral area 22 |
|  | MTG, Middle Temporal Gyrus | MTG_L(R)_4_1 | A21c, caudal area 21 |
|  |  | MTG_L(R)_4_2 | A21r, rostral area 21 |
|  |  | MTG_L(R)_4_3 | A37dl, dorsolateral area37 |
|  |  | MTG_L(R)_4_4 | aSTS, anterior superior temporal sulcus |
|  | ITG, Inferior Temporal Gyrus | ITG_L(R)_7_1 | A20iv, intermediate ventral area 20 |
|  |  | ITG_L(R)_7_2 | A37elv, extreme lateroventral area37 |
|  |  | ITG_L(R)_7_3 | A20r, rostral area 20 |
|  |  | ITG_L(R)_7_4 | A20il, intermediate lateral area 20 |
|  |  | ITG_L(R)_7_5 | A37vl, ventrolateral area 37 |
|  |  | ITG_L(R)_7_6 | A20cl, caudolateral of area 20 |
|  |  | ITG_L(R)_7_7 | A20cv, caudoventral of area 20 |
|  | FuG, Fusiform Gyrus | FuG_L(R)_3_1 | A20rv, rostroventral area 20 |
|  |  | FuG_L(R)_3_2 | A37mv, medioventral area37 |
|  |  | FuG_L(R)_3_3 | A37lv, lateroventral area37 |
|  | PhG, Parahippocampal Gyrus | PhG_L(R)_6_1 | A35/36r, rostral area 35/36 |
|  |  | PhG_L(R)_6_2 | A35/36c, caudal area 35/36 |
|  |  | PhG_L(R)_6_3 | TL, area TL (lateral PPHC, posterior parahippocampal gyrus) |
|  |  | PhG_L(R)_6_4 | A28/34, area 28/34 (EC, entorhinal cortex) |
|  |  | PhG_L(R)_6_5 | TI, area TI(temporal agranular insular cortex) |
|  |  | PhG_L(R)_6_6 | TH, area TH (medial PPHC) |
|  | pSTS, posterior Superior Temporal Sulcus | pSTS_L(R)_2_1 | rpSTS, rostroposterior superior temporal sulcus |
|  |  | pSTS_L(R)_2_2 | cpSTS, caudoposterior superior temporal sulcus |
| **Parietal Lobe** | SPL, Superior Parietal Lobule | SPL_L(R)_5_1 | A7r, rostral area 7 |
|  |  | SPL_L(R)_5_2 | A7c, caudal area 7 |
|  |  | SPL_L(R)_5_3 | A5l, lateral area 5 |
|  |  | SPL_L(R)_5_4 | A7pc, postcentral area 7 |
|  |  | SPL_L(R)_5_5 | A7ip, intraparietal area 7(hIP3) |
|  | IPL, Inferior Parietal Lobule | IPL_L(R)_6_1 | A39c, caudal area 39(PGp) |
|  |  | IPL_L(R)_6_2 | A39rd, rostrodorsal area 39(Hip3) |
|  |  | IPL_L(R)_6_3 | A40rd, rostrodorsal area 40(PFt) |
|  |  | IPL_L(R)_6_4 | A40c, caudal area 40(PFm) |
|  |  | IPL_L(R)_6_5 | A39rv, rostroventral area 39(PGa) |
|  |  | IPL_L(R)_6_6 | A40rv, rostroventral area 40(PFop) |
|  | Pcun, Precuneus | PCun_L(R)_4_1 | A7m, medial area 7(PEp) |
|  |  | PCun_L(R)_4_2 | A5m, medial area 5(PEm) |
|  |  | PCun_L(R)_4_3 | dmPOS, dorsomedial parietooccipital sulcus(PEr) |
|  |  | PCun_L(R)_4_4 | A31, area 31 (Lc1) |
|  | PoG, Postcentral Gyrus | PoG_L(R)_4_1 | A1/2/3ulhf, area 1/2/3(upper limb, head and face region) |
|  |  | PoG_L(R)_4_2 | A1/2/3tonIa, area 1/2/3(tongue and larynx region) |
|  |  | PoG_L(R)_4_3 | A2, area 2 |
|  |  | PoG_L(R)_4_4 | A1/2/3tru, area1/2/3(trunk region) |
| **Insular Lobe** | INS, Insular Gyrus | INS_L(R)_6_1 | G, hypergranular insula |
|  |  | INS_L(R)_6_2 | vIa, ventral agranular insula |
|  |  | INS_L(R)_6_3 | dIa, dorsal agranular insula |
|  |  | INS_L(R)_6_4 | vId/vIg, ventral dysgranular and granular insula |
|  |  | INS_L(R)_6_5 | dIg, dorsal granular insula |
|  |  | INS_L(R)_6_6 | dId, dorsal dysgranular insula |
| **Limbic Lobe** | CG, Cingulate Gyrus | CG_L(R)_7_1 | A23d, dorsal area 23 |
|  |  | CG_L(R)_7_2 | A24rv, rostroventral area 24 |
|  |  | CG_L(R)_7_3 | A32p, pregenual area 32 |
|  |  | CG_L(R)_7_4 | A23v, ventral area 23 |
|  |  | CG_L(R)_7_5 | A24cd, caudodorsal area 24 |
|  |  | CG_L(R)_7_6 | A23c, caudal area 23 |
|  |  | CG_L(R)_7_7 | A32sg, subgenual area 32 |
| **Occipital Lobe** | MVOcC*,* MedioVentral Occipital Cortex | MVOcC _L(R)_5_1 | cLinG, caudal lingual gyrus |
|  |  | MVOcC _L(R)_5_2 | rCunG, rostral cuneus gyrus |
|  |  | MVOcC _L(R)_5_3 | cCunG, caudal cuneus gyrus |
|  |  | MVOcC _L(R)_5_4 | rLinG, rostral lingual gyrus |
|  |  | MVOcC _L(R)_5_5 | vmPOS,ventromedial parietooccipital sulcus |
|  | LOcC, lateral Occipital Cortex | LOcC_L(R)_4_1 | mOccG, middle occipital gyrus |
|  |  | LOcC _L(R)_4_2 | V5/MT+, area V5/MT+ |
|  |  | LOcC _L(R)_4_3 | OPC, occipital polar cortex |
|  |  | LOcC_L(R)_4_4 | iOccG, inferior occipital gyrus |
|  |  | LOcC _L(R)_2_1 | msOccG, medial superior occipital gyrus |
|  |  | LOcC _L(R)_2_2 | lsOccG, lateral superior occipital gyrus |
| **Subcortical Nuclei** | Amyg, Amygdala | Amyg_L(R)_2_1 | mAmyg, medial amygdala |
|  |  | Amyg_L(R)_2_2 | lAmyg, lateral amygdala |
|  | Hipp, Hippocampus | Hipp_L(R)_2_1 | rHipp, rostral hippocampus |
|  |  | Hipp_L(R)_2_2 | cHipp, caudal hippocampus |
|  | BG, Basal Ganglia | BG_L(R)_6_1 | vCa, ventral caudate |
|  |  | BG_L(R)_6_2 | GP, globus pallidus |
|  |  | BG_L(R)_6_3 | NAC, nucleus accumbens |
|  |  | BG_L(R)_6_4 | vmPu, ventromedial putamen |
|  |  | BG_L(R)_6_5 | dCa, dorsal caudate |
|  |  | BG_L(R)_6_6 | dlPu, dorsolateral putamen |
|  | Tha, Thalamus | Tha_L(R)_8_1 | mPFtha, medial pre-frontal thalamus |
|  |  | Tha_L(R)_8_2 | mPMtha, pre-motor thalamus |
|  |  | Tha_L(R)_8_3 | Stha, sensory thalamus |
|  |  | Tha_L(R)_8_4 | rTtha, rostral temporal thalamus |
|  |  | Tha_L(R)_8_5 | PPtha, posterior parietal thalamus |
|  |  | Tha_L(R)_8_6 | Otha, occipital thalamus |
|  |  | Tha_L(R)_8_7 | cTtha, caudal temporal thalamus |
|  |  | Tha_L(R)_8_8 | lPFtha, lateral pre-frontal thalamus |

**FIGURE S1 Result of correction for multiple comparisons based on the network-based statistic (NBS).** Distribution of connections with significant differences based on (A) morphological brain network; (B) structural brain network; (C) functional brain network. In present study, NBS was used to conduct multiple comparisons. A corrected P-value was calculated for each component using the null distribution of the maximal connected component size, which was empirically derived using a nonparametric permutation approach (5000 permutations). P < 0.01 indicated a significant difference. Our results indicated that the connections with significant differences between SCD and HCs in morphological brain network were mainly distributed in Cingulate Gyrus (CG), Inferior Parietal Lobule (IPL), Insular Gyrus (INS), Middle Frontal Gyrus (MFG), Superior Frontal Gyrus (SFG), and Paracentral Lobule (PCL). Meanwhile, the connections with significant differences in structural brain network were mainly distributed in Thalamus (Tha), Superior Temporal Gyrus (STG), Superior Frontal Gyrus (SFG), and Precentral Gyrus (PrG). Furthermore, it is worth noting that compared with the morphological and structural networks, the functional network presented a larger number and wider range of connections with significant differences. These connections mainly distributed between the cortex (e.g., Middle Frontal Gyrus [MFG], Postcentral Gyrus [PoG], Superior Parietal Lobule [SPL]) and the limbic system (e.g., hippocampus [Hipp], Parahippocampal Gyrus [PhG], Cingulate Gyrus [CG]) and the subcutaneous nucleus (e.g., Basal Ganglia [BG], Amygdala [Amyg], and Thalamus [Tha]). The colors were randomly generated to differentiate regions of interests (ROIs). The figure was conducted with a MATLAB function, circularGraph, shared by Paul Kassebaum (http://www.mathworks.com/matlabcentral/fileexchange/48576-circulargraph).


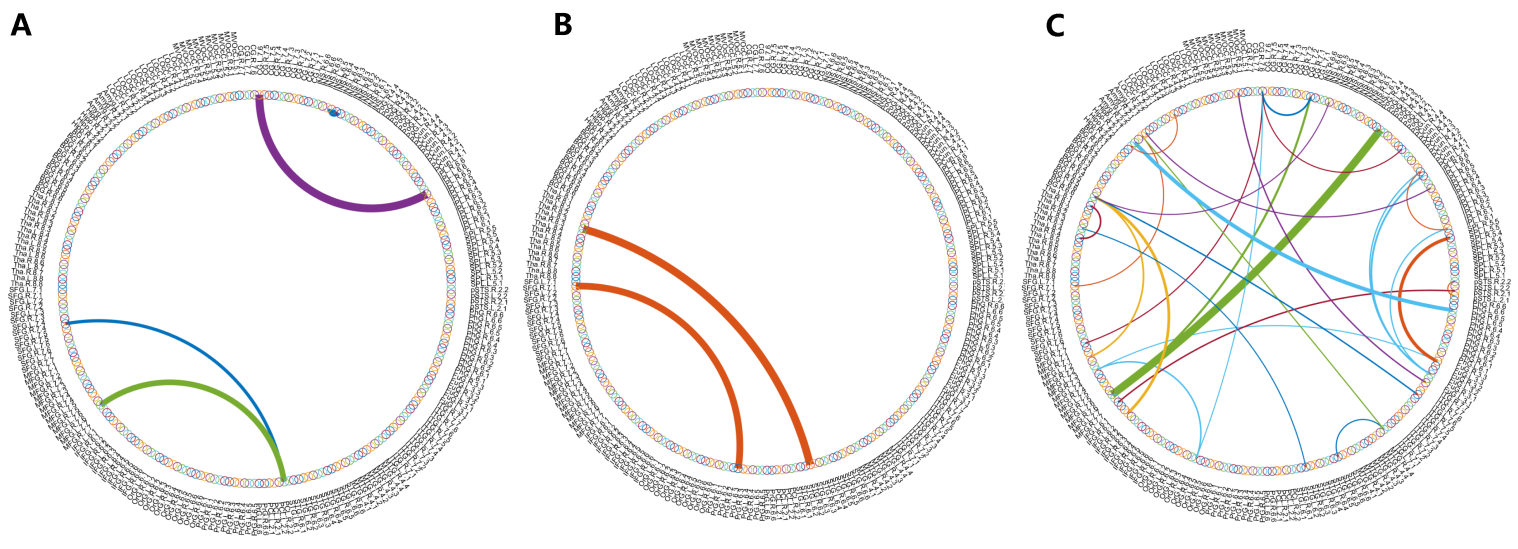


**FIGURE S2** Morphological, structural, and functional network matrices for healthy controls at the group level based on the AAL 90 template. AAL, automated anatomical labelling atlas


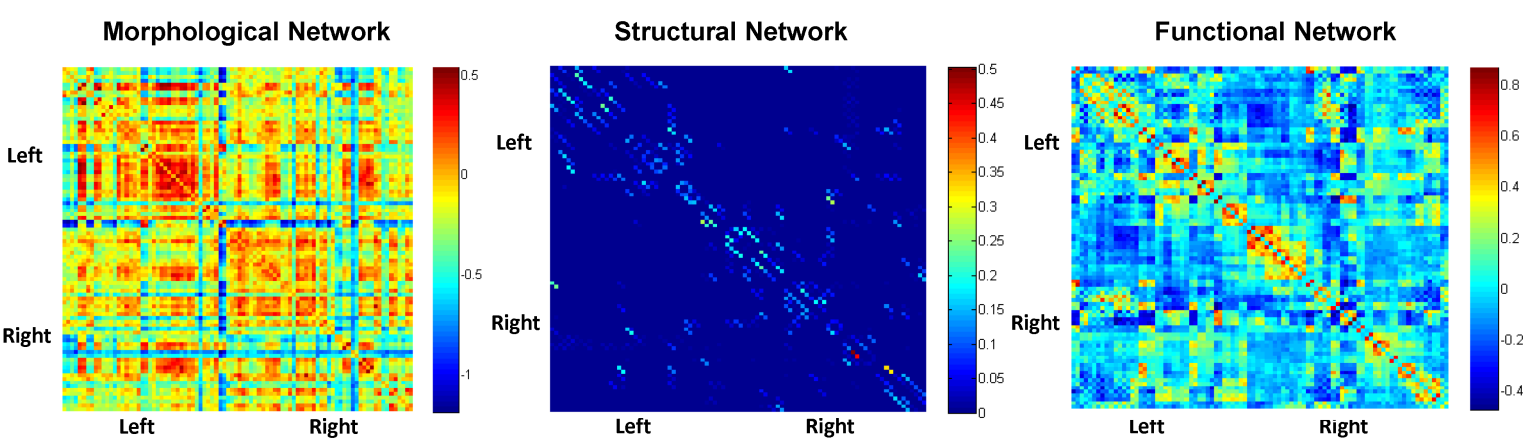


**FIGURE S3** Hub nodes of the SCD and HC groups in different brain networks based on the AAL 90 template. (A) Hub nodes of SCD in morphological, structural and functional brain network based on sMRI, DTI and fMRI; (B) Hub nodes of HC in morphological, structural and functional brain network based on sMRI, DTI and fMRI. The hub nodes were mapped on the ICBM 152 template with the BrainNetViewer package (http://nitrc.org/projects/bnv/). AAL, automated anatomical labelling atlas; sMRI, structural magnetic resonance imaging; DTI, diffusion tensor imaging; fMRI, functional magnetic resonance imaging; SCD, subjective cognitive decline; HC, healthy control.


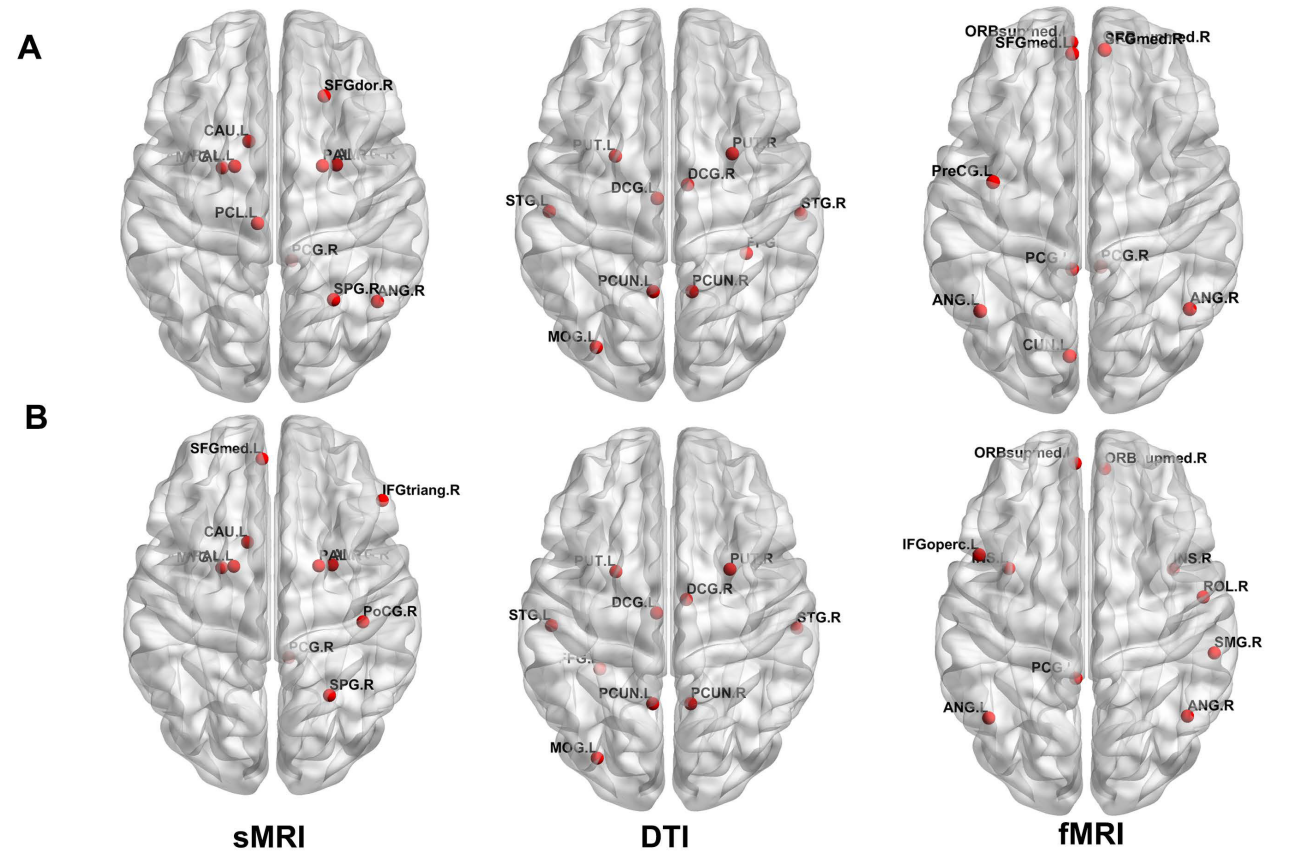


**FIGURE S4** Distribution of consensus connections identified by different modalites based on the AAL 90 template. (A) Morphological brain network based on sMRI; (B) Structural brain network based on DTI; (C) Functional brain network based on fMRI. The consensus connections were mapped on the ICBM 152 template with the BrainNetViewer package (http://nitrc.org/projects/bnv/). Red and blue lines represent the increased and decreased connectivity weight of the SCD group, respectively. AAL, automated anatomical labelling atlas; sMRI, structural magnetic resonance imaging; DTI, diffusion tensor imaging; fMRI, functional magnetic resonance imaging.


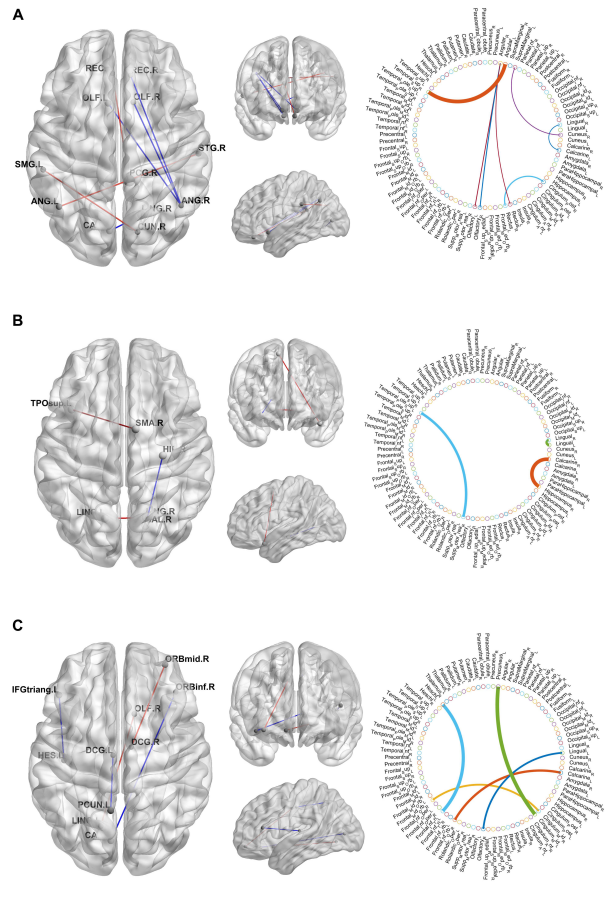


**FIGURE S5** Receiver operating characteristic (ROC) of classifications of different modalities based on the AAL 90 template. AAL, automated anatomical labelling atlas; ROC, receiver operating characteristic


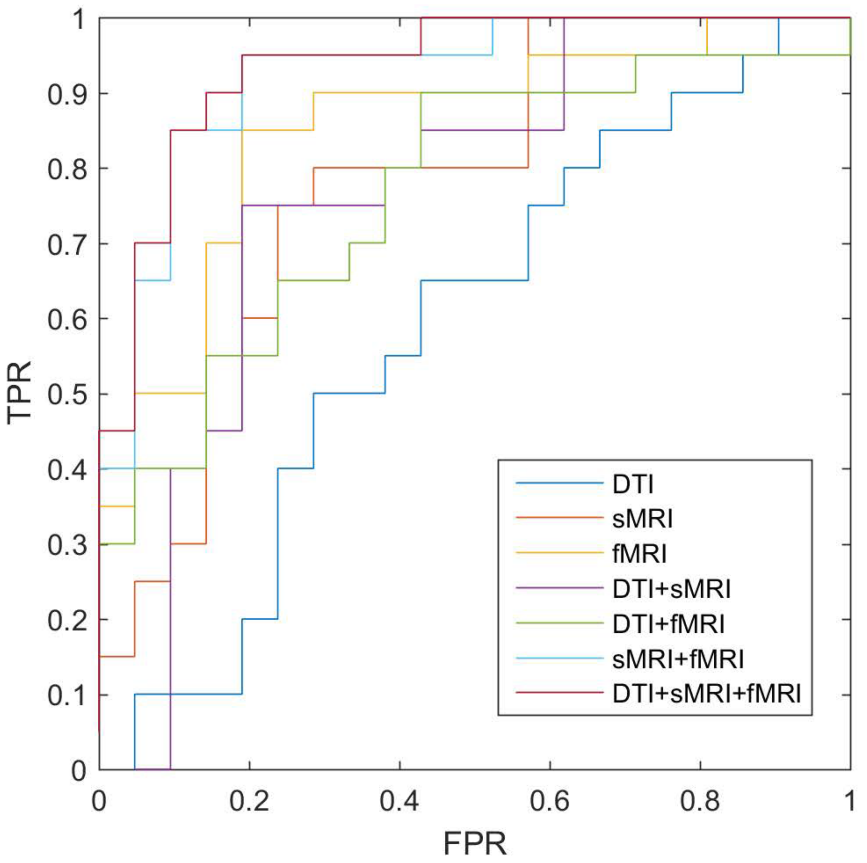

Supplement: Supplementary file 1 [file Table_1.docx]
