# Supplementary figures and images for: Morphological, Structural, and Functional Networks Highlight the Role of the Cortical-Subcortical Circuit in Individuals With Subjective Cognitive Decline
Source: Front Aging Neurosci. 2021 Jul 9;13:688113. doi: 10.3389/fnagi.2021.688113 (PMC8299728; doi:10.3389/fnagi.2021.688113)

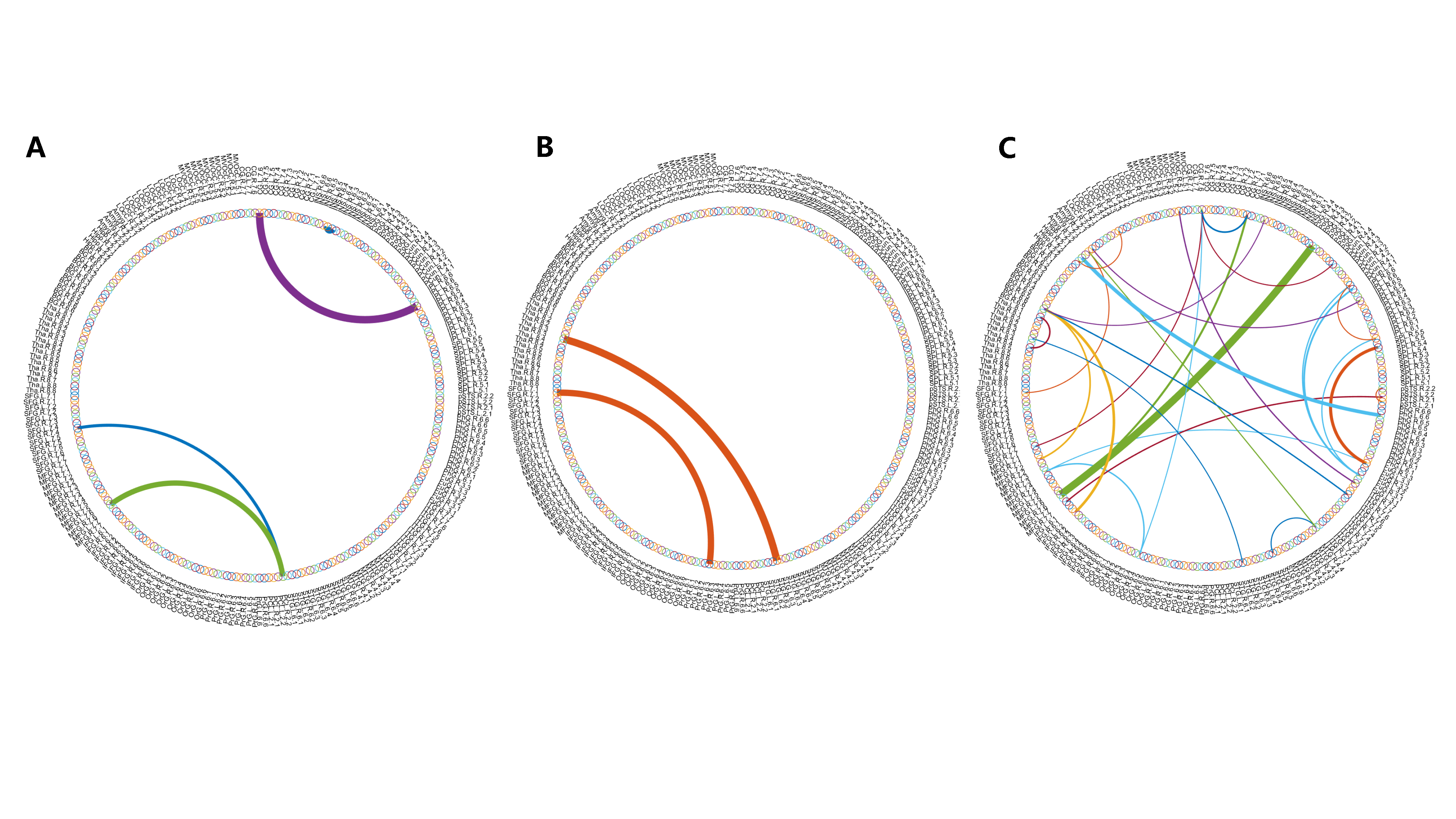

Supplement: Supplementary file 2 [file Image_1.TIF]

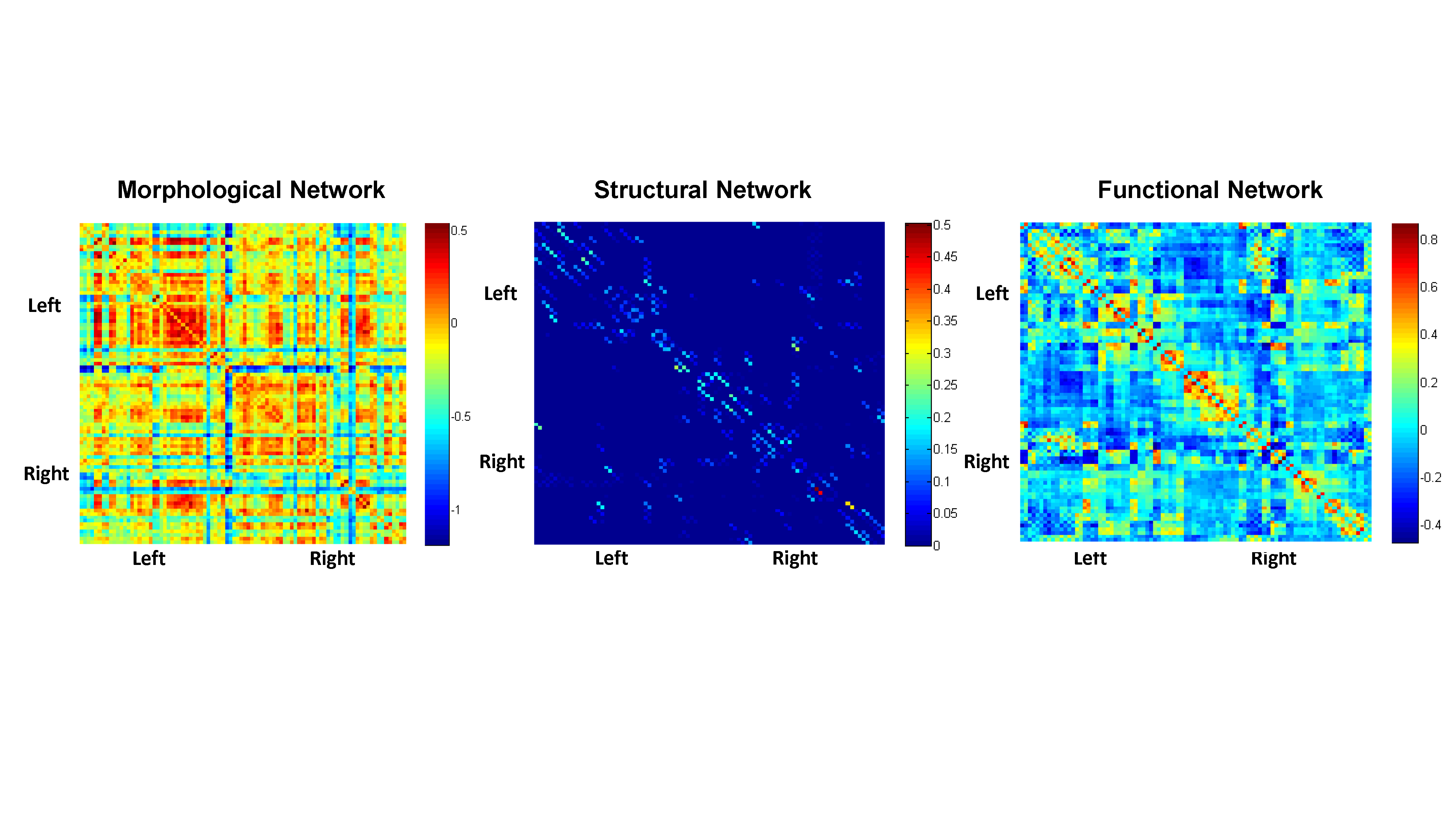

Supplement: Supplementary file 3 [file Image_2.TIF]

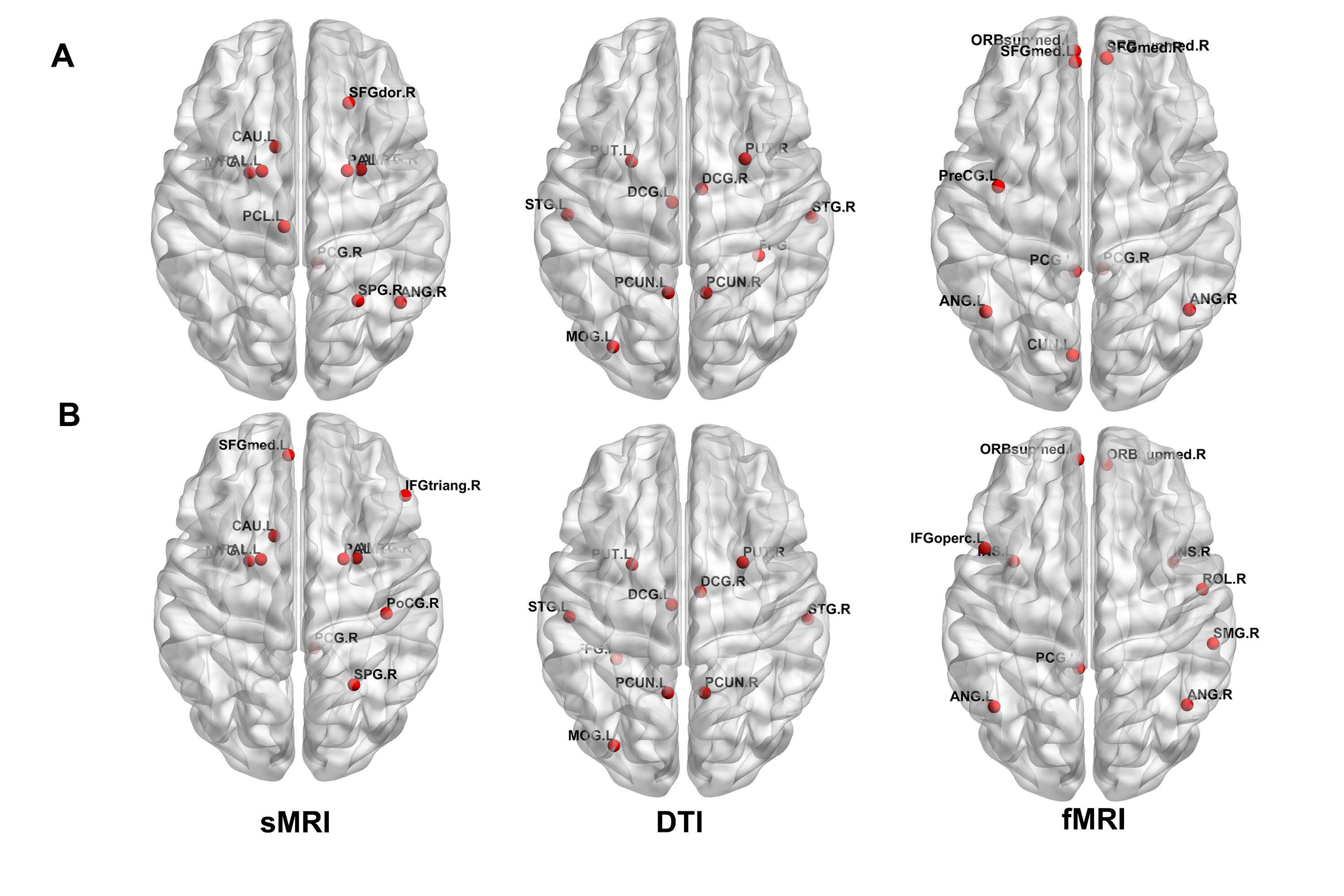

Supplement: Supplementary file 4 [file Image_3.TIF]

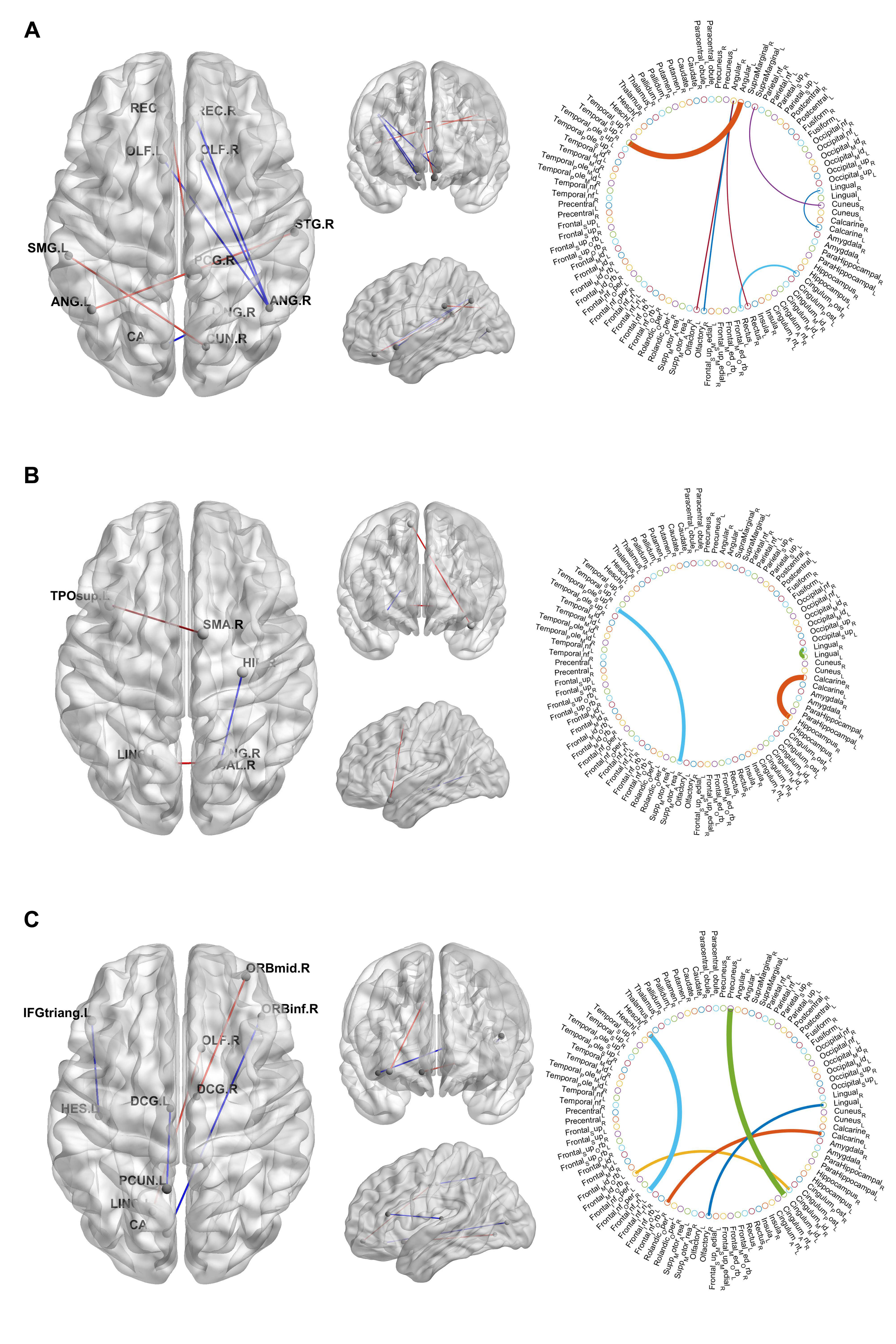

Supplement: Supplementary file 5 [file Image_4.TIF]

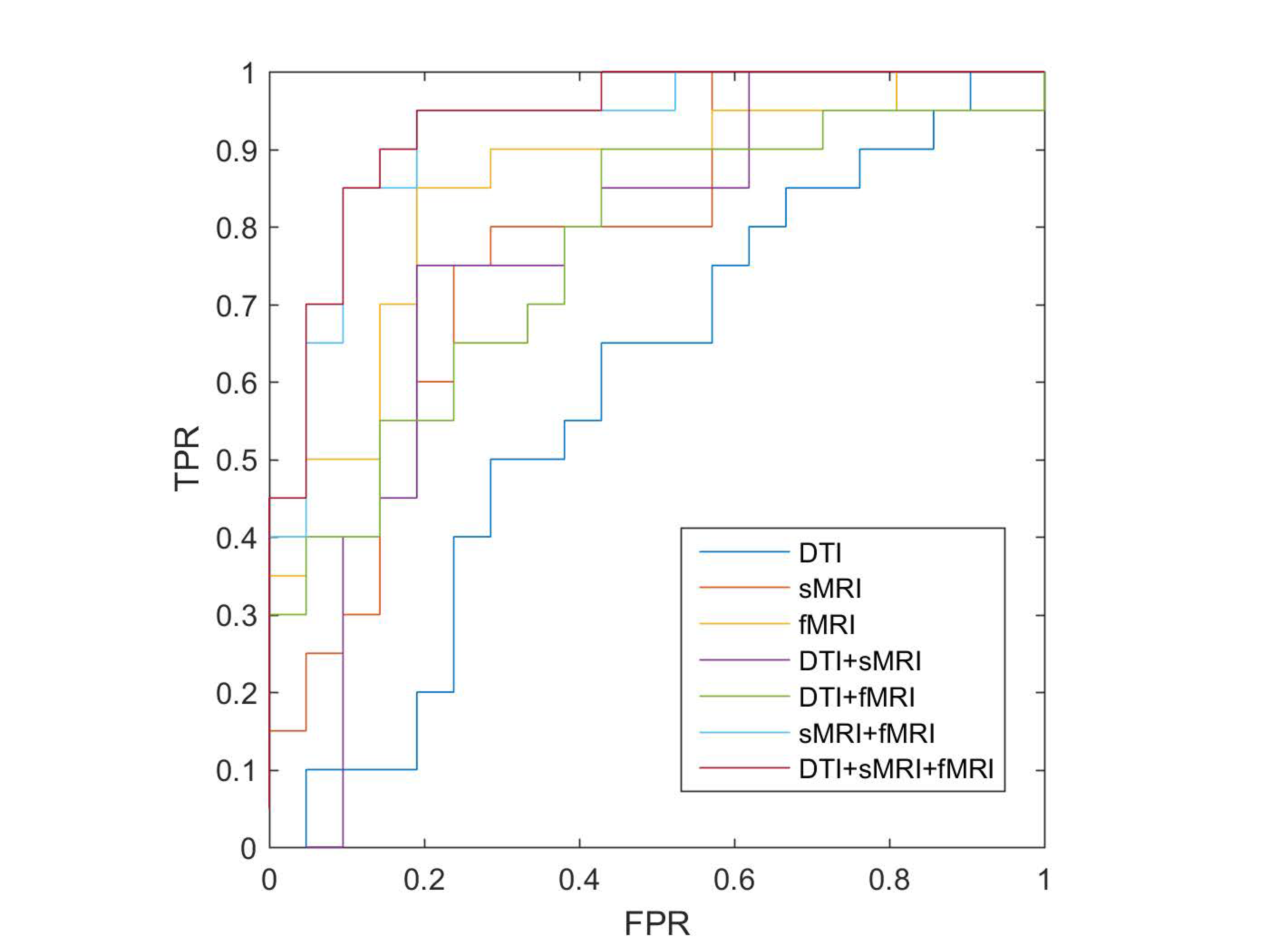

Supplement: Supplementary file 6 [file Image_5.TIF]
